# A visual analytic method to investigate patterns and structures of missing data

**DOI:** 10.1371/journal.pone.0355400

**Published:** 2026-08-07

**Authors:** Roy A. Ruddle

**Affiliations:** 1 School of Computer Science, University of Leeds, Leeds, United Kingdom; 2 Leeds Institute for Data Analytics, University of Leeds, Leeds, United Kingdom; Lodz University of Technology: Politechnika Lodzka, POLAND

## Abstract

Datasets often have missing values. Understanding the locations and underlying reasons (missing data patterns and structures, respectively) is important to avoid analysis bias or building inappropriate models. However, investigating missing data patterns and structures presents significant challenges. To address them we developed a novel visual analytic method, which combines new purity metrics for three core types of pattern (block, monotone and disjoint) with seven different visualizations in an iterative workflow. We also conducted two case studies, one with a UCI Machine Learning Repository dataset and the other with a 21 million record/389 variable hospital dataset that had many interwoven missingness patterns. In the UCI case study, the method’s purity metrics and heatmaps revealed a rare data quality issue that affected 0.08% of records, but was not apparent when an existing correlation-based approach was used. Our method also implements perceptual discontinuity to ensure that small values are visible in bar charts, and that showed that two variables were missing values in 0.02% and 0.3% of records, respectively, rather than being complete as reported in the original UCI paper. The hospital dataset contained 595,171 unique combinations of missing values, but that overwhelming number was reduced to 57 comprehensible patterns by iteratively using purity metrics and heatmaps with the three core pattern types in our method. That revealed insights that ranged from uncovering rare and common data quality issues, to finding data dictionary errors, and making explicit relationships between groups of variables that may otherwise be hidden. The method also provides an explanation graph, which aids reproducibility by documenting the order in which patterns were found and acts as a tangible artifact that should help communicate the findings to stakeholders.

## Introduction

Missing data is the most common data quality issue that analysts, data scientists and model developers encounter. Missing data can lead to bias, invalid findings, inaccurate parameter estimations, reduce the statistical power of a model, reduced generalizability or prevent the use of a given model [1–3]. The main approaches for understanding missing data are counting the number of values that are missing from each variable, or characterizing the mechanism that is thought to underlie each variable’s missingness (missing not at random (MNAR), etc. [4]).

The term “missing data pattern” concerns the location of missing values across the variables in a dataset, whereas a “structure of missingness” occurs where a given pattern is associated with other factors or attributes of the data [5]. Nine grand challenges have been identified to address the effects of structured missingness in data [5] and, in the present research, we address *Challenge 2: Exploring the geometry of structured missingness*. Key aspects of that challenge are the added difficulty of detecting patterns of missingness that are thinly spread, the importance of higher-order rather than just pairwise patterns, and the need for structured missingness methods to work effectively with highly multivariate datasets.

This paper describes a novel visual analytic method that enables users to comprehensively investigate patterns and structures of missing values in tabular data, whether they involve a specific group of variables or are interwoven across multiple groups. To create the method we: (a) propose three patterns (block, monotone and disjoint) to act as the building blocks of missingness structures, (b) define new metrics for quantifying the purity of each pattern, (c) provide a set of visualizations to help users use the metrics to identify important patterns, and (d) create an explanation graph and summary to show how the patterns/structures interrelate and bring reproducibility and rigor to the investigation. The paper’s final contribution is two case studies that show the types of insight the method can provide. The method is implemented in the *vizdataquality* Python package, which is available on PyPI [6].

## Related work

Data can be missing for many reasons. During data collection, each item of data may be mandatory, conditional (required under certain conditions) or optional [3]). In other situations data may be missing by design, there may be procedural or recording errors (e.g., a sensor failure), unexpected events (e.g., a power cut) or the data may refer to items with heterogeneous characteristics [1,2,5]. With longitudinal data the attributes that are collected often evolve as organizations’ requirements change [7] or organizations merge [8]. In longitudinal studies, participant dropout progressively reduces the cohort size and risks increasing bias [1,9]. When combining data from different sources, they may not have recorded the same variables [10], at the same granularity [11] or be misaligned [12].

The most common way of handling missing data is to either exclude records or impute the missing values [13]. Exclusion always reduces the amount of information in a dataset and can cause bias [3]. The same is true for basic imputation methods such as using the mean value of a variable [14]. Multiple imputation aims to incorporate uncertainty about the imputed values [3], but still has a variety of potential pitfalls, including situations where the data violates important assumptions (e.g., being normally distributed) or the underlying reasons for the missing values mean they cannot be accounted for by the remaining variables [15]. Sometimes so many records are missing that false conclusions are drawn (e.g., about mortality in a children’s heart unit, leading to its closure for a period of time [8]) and only rectified after further data is obtained. Lastly, some models and machine learning algorithms handle missing values (e.g., lightGBM [16]) but others cannot (e.g., the Scikit-learn random forest classifier).

The remainder of this section is divided into three parts, which briefly review terminology, visualization approaches for investigating missing data, and methods for improving reproducibility and rigor. Together, these provide a foundation for our research into a visual analytic method that enables people to comprehensively investigate patterns and structures of missing data.

### Terminology

When analyzing missing data it is important to differentiate between mechanisms and patterns [5]. The mechanisms are the processes by which values become missing, and the three best-known mechanisms are missing completely at random (MCAR), missing at random (MAR) and missing not at random (MNAR) [4]. By contrast, the way that missing values are located in data defines the missing data patterns, and when an associated explanation is provided a pattern may be termed a structure. Mechanisms and patterns are two fundamentally different characterizations of missing data, and it is well known that the same pattern can be produced by a variety of different mechanisms or a mixture of them (e.g., MCAR, MAR and/or MNAR).

Researchers have used a number of different terms to describe missing data patterns, including unit nonresponse, block, planned, latent variable, univariate, monotone and joint missingness [5,17,18]. Some work uses the terms non-monotone or general to describe any pattern that is not monotone [19].

The latent variable pattern is an example of the “empty column” data quality issue [20]. By contrast, a univariate pattern is where a single variable is partially missing and all of the other variables in a dataset are complete.

**Fig 1:**
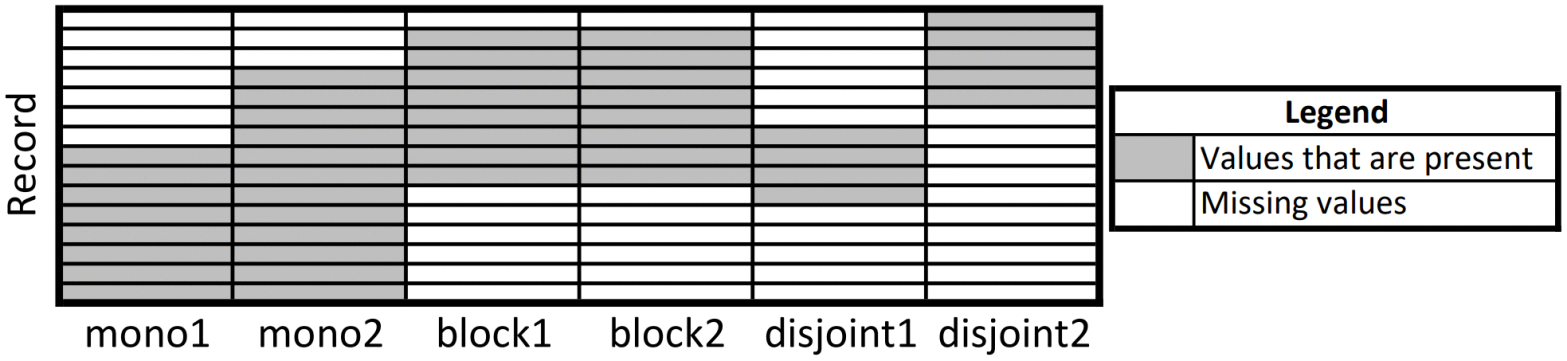
A 15-record dataset that contains pure monotone (variables mono1 & mono2) block (variables block1 & block2) and disjoint patterns of missing values (variables disjoint1 & disjoint2).

Joint missingness refers to where two or more variables are missing together, with the best-known example being a monotone pattern. A monotone pattern looks like a staircase (Fig 1), where each variable is missing successively more values, and that pattern is common where sets of similar variables are provided (e.g., up to 20 variables for a patient’s diagnoses in hospital data) [21] or longitudinal studies where some participants drop out [17,22]. Another type of joint missingness is a block pattern (Fig 1; also termed unit nonresponse [17]), which occurs where the same variables are missing in a set of records [23].

The planned missing pattern [17,24] would be termed “disjoint” in set terminology, because each record only belongs to the set of records that is missing one of the variables in the pattern (Fig 1).

Some researchers transform data by setting missing and present values equal to zero and one, respectively, and then calculating Pearson correlations to identify patterns between pairs of variables [25]. If two variables were always missing together (a block pattern) then they would have a correlation of 1.0, whereas if the variables were only missing separately (a disjoint pattern) then they would have a correlation of −1.0. However, real data tends to contain some noise so the patterns are rarely pure, which is problematic because the correlation coefficient is affected by the amount of noise and the overall proportion of values that are missing. Also, correlations are not suitable for detecting monotone patterns because pure patterns have a correlation coefficient (*r*) in the range 0.0 < *r* < 1.0.

It is not uncommon for datasets to contain numerous, overlapping patterns of missing values, and that is particularly the case for large, multivariate datasets [26]. However, the limitations of current taxonomies and deficiencies of current tools make it difficult for users to disentangle those patterns and, therefore, to identify the underlying structures of missingness that lead to those patterns [5]. Understanding those structures influences a user’s choice of imputation method [3].

### Visualization approaches

One approach to visualizing missing data embeds information about missing values within other visualizations. Examples include stating the number of null data points [20], adding marks or histograms to the axes of a chart [3,27], giving missing data a special value [18], and highlighting missing values [12].

A different approach is to visualize a “quality space” [28] where the primary focus is the missing data. A basic example, found in many tools and libraries, is using a bar chart to show the number of values that are missing from each variable [13,29,30]. A scalability issue is that complete variables large datasets may be indistinguishable from those with only a small percentage of missing values, because bars for the latter would be too short to be visible. However, that is easily remedied with software that implements perceptual discontinuity [31].

More sophisticated examples for visualizing combinations of missing variables are matrix plots (sometimes called a ”missingness map”) [3,9,18,23,25,32], lasagna plots [33], upset plots [34–36] and heatmaps [3,21,25,37,38].

A variety of types of plots may be used to visualize associations of missing values with known values of another variable (e.g., a histogram, stacked bar chart, spinogram or spine plot). Parallel coordinates have been suggested for visualizing those associations for multiple variables, and mosaic plots (a type of treemap) for associations with pairs of categorical variables [3].

There are some approaches that are used to visualize combinations of missing variables but each of these has fundamental limitations. Matrix plots (sometimes called a “missingness map”) require a separate column for each variable as well as a separate row for each record [3,9,18,23,25,32,38] so are only suitable for small datasets not ones containing many thousands or millions of records like those used in our two case studies and many other real-world datasets. Lasagna plots are based on matrix plots [33] and have some advantages for visualizing longitudinal patterns in missing data, but as a result are not designed for multivariate data. Upset plots [34–36] and heatmaps [3,25,37] require a separate column for each variable (like matrix plots) but are more scalable because the rows show unique combinations of missing values rather than each record. In practice, upset plots and heatmaps are considered suitable for visualizing up to 100 combinations of missing values from 25–65 variables [34], limits that are similar to those reported by a state-of-the-art review [39]. One rare exception is a study that visualized 4371 combinations of missing value from 86 variables in a heatmap, but that required a six-monitor, 24 megapixel display that measured 1.9 x 0.9 meters [21].

Missing value combinations are determined by treating all of the records that are missing a given variable as a set and then computing the exclusive set intersections (ESIs). However, highly multivariate datasets tend to have many interwoven patterns of missingness, which act as a multiplier for the number of ESIs and this may produce noisy upset plots/heatmaps. An open question is what techniques would alleviate that noise? A further barrier for some missing value software is inefficient data structures that necessitate an excessive amount of RAM memory [40].

### Reproducibility and rigor

A general missing data workflow can be divided into four steps: (1) calculate summary statistics (the number of missing values in each variable), (2) investigate combinations of variables that are missing together, (3) apply remedial action (e.g., exclude records or impute missing values), and (4) evaluate that remedial action [3,7]. Analysts and data scientists often implement such workflows in scripts, Jupyter Notebooks or R Markdown, and a similar workflow has been provided in visual analytic tools such as VIVID [9].

Reproducibility depends on capturing provenance and ”data” is the type of provenance information that specifically concerns the history of a dataset, including transformations brought about by data quality issues [41]. A graph structure is well-suited to capturing provenance information [42] and has been implemented in a variety of visualization tools [43–45].

To our knowledge, no previous research has applied such techniques to the investigation of patterns and structures of missing data. Therefore, to bring reproducibility and rigour to such investigations our visual analytic method provides an explanation graph that records: (a) the order in which a user identified each missing data pattern in a dataset, (b) ways in which those patterns interrelate in terms of shared columns and/or combinations of missing variables, (c) descriptions of underlying structures in those patterns, and (d) an explanation summary. The summary helps users to identify missingness that is still unexplained or only partially explained, and ensure that nothing is overlooked.

## Visual analytic method

Drawing on the “exploring the geometry of structured missingness” grand challenge [5] and many years of personal experience dealing with missing data, we identified five core requirements for methods to enable the investigation of missing data patterns and structures:

- **R1:** Reveal both rare and common patterns
- **R2:** Show patterns involving any number of variables
- **R3:** Effective with highly multivariate datasets
- **R4:** Capable of comprehensively accounting for all of the missingness in a dataset
- **R5:** Document the investigation in a reproducible manner

Regarding **R1**, although existing methods are capable to detecting common patterns of missing data they are not effective for rare patterns (e.g., correlations are weak for rare missing data patterns; see below). **R2** requires the capability to show patterns involving any number of variables, but correlations only reveal pairwise patterns and, of the visualization approaches described above, bar charts show missing value counts not multi-variable patterns (e.g., [29]) and missingness maps are only suitable for small datasets because a separate row is required for each record (e.g., [3]). Large, highly multivariate datasets (**R3**) can contain a huge number of combinations of missing value (e.g., half a million combinations in our NHS hospital admissions case study) and that far exceeds the limits of current visualizations such as upset plots and combination heatmaps (see Visualization approaches, above). Regarding **R4**, as far as we are aware no existing methods comprehensively account for all of the missingness in a dataset or provide a framework such as our Explanation graph (see below) to support reproducibility (**R5**).

This section describes the design of the computational, visualization and explanation graph components of our method, which has been implemented in the *vizdataquality* Python package [6]. The “Diabetes 130-US Hospitals for Years 1999-2008” dataset [46] from the UCI Machine Learning Repository is used to illustrate the method.

### Computation

The first step for investigating missing data patterns is to calculate the number of times each variable is missing and the combinations of variables that are missing together [3]. The number of combinations cannot be greater than the number of records in a dataset and in practice is orders of magnitude fewer, helping the method scale to highly multivariate datasets (Requirement **R3**). For example, in the Diabetes dataset there are only 54 combinations of missing values in the 101,766 records, and in a 16 million electronic health records and 86 variables there were only 4371 combinations of missing values, despite the fact that almost every record was missing at least one value [31].

Applying set theory to missing data, a set may be created for the values that are missing from each variable. A set intersection is then created for records that are missing the same variables, whether that be only one or any number of variables (Requirement **R2**). Our method uses a pair of data structures: Intersections are contained in a dataframe with one row per intersection and one column per variable, and a second dataframe contains one row per dataset record and stores the ID of that record’s intersection. These data structures are very memory efficient (e.g., 15−20 times smaller than the original dataset in our two case studies), which is also important for highly multivariate datasets (**R3**). Our vizdataquality package also includes functions to export and import the data structures.

The method is based on three core missing data patterns (block, monotone and disjoint). Previous research and data quality literature typically describes patterns in a clean fashion, for example, variables that record up to 20 diagnoses in UK hospitals (a monotone pattern; each patient should have a primary diagnosis, but only patients with the most complex health conditions will have values for all 20 diagnosis variables), the genes included in cancer tests (a block pattern produced by different assays being used in genomic tests) or an answer to one survey question meaning that certain other questions were omitted (disjoint pattern) [5,17,21].

In practice missing data patterns are often not perfect (e.g., 1776 out of 16 million hospital records not adhering to the expected monotone pattern because they contained gaps in patients’ diagnoses [21]). A common way of measuring the purity of a pattern is to set each missing value to zero and each present value to one, and then calculate the Pearson correlation of pairs of variables. Perfect block and disjoint patterns have a correlation equal to 1.0 and −1.0, respectively. However, there are disadvantages for quantifying impure patterns because the value of a correlation may change with the number of records in a dataset and is not well-suited to imbalanced data.

**Fig 2:**
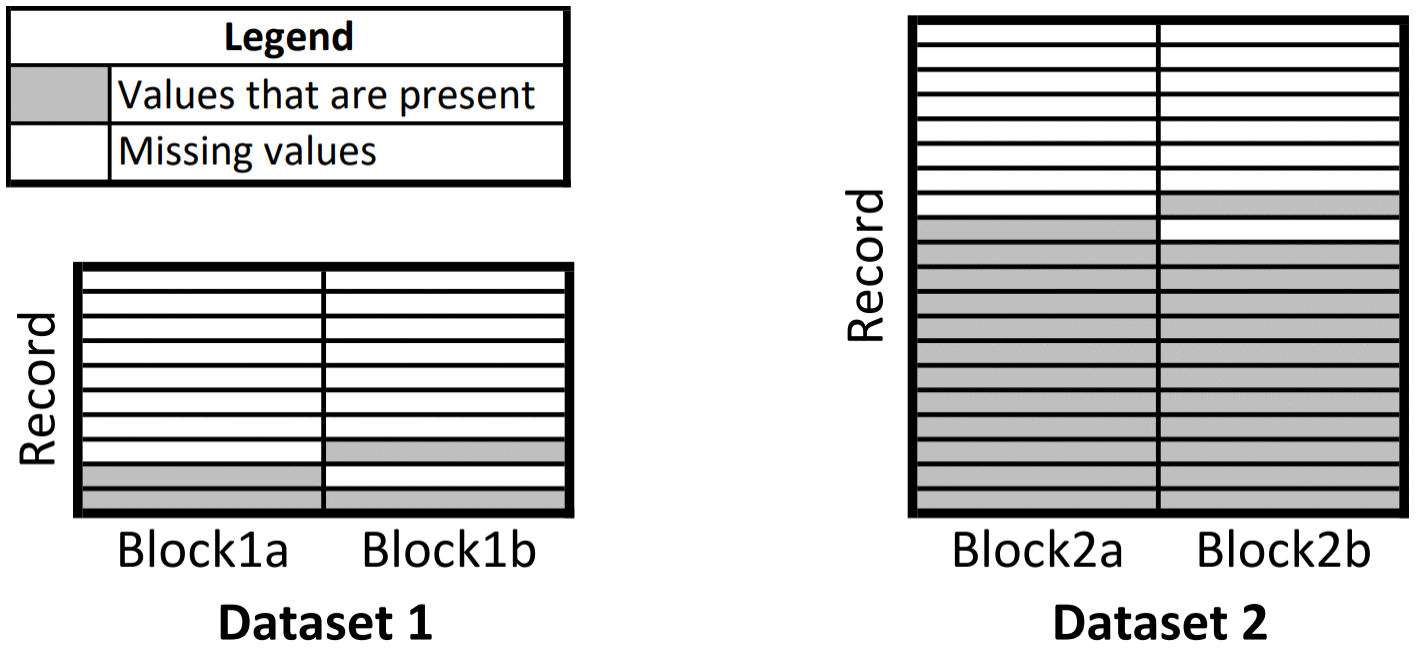
Two datasets with the same missing values in an impure block pattern, but a different number of present values.

Some missing data patterns are common but others are rare, and often it is the rare patterns that are unexpected and unknown to users [21]. Some researchers calculate Pearson correlations to identify pairs of variables that tend to have missing values together (a block pattern) [25]. However, correlations are calculated from all records (missing and present values set equal to zero and one, respectively) so the approach is poorly suited to detecting rare patterns of missingness (Requirement **R1**). That is best illustrated using an example. Both of the datasets shown in Fig 2 have an identical number and pattern of missing values, but Dataset 2 contains twice as many records and all of those extra records are complete, so Dataset 1 has a much weaker correlation (r(8) = .375) than Dataset 2 (r(18) = .792). A solution is to calculate the purity of a block pattern solely from the incomplete records, as shown in Equation 1. Datasets 1 and 2 have the same block pattern purity of *B* = .778.


$$\begin{array}{c} B=M_{AB}/(M_{AB}+M_{A}+M_{B}) \end{array}$$ (1)

where

$M_{AB}$ is the number of records missing values for both variables A and B

$M_{A}$ is the number of records missing values for just variable A

$M_{B}$ is the number of records missing values for just variable B

A similar approach is taken to calculate the purity *D* of a disjoint pattern (see Equation 2):


$$\begin{array}{c} D=(M_{A}+M_{B})/(M_{AB}+M_{A}+M_{B}) \end{array}$$ (2)

One way of calculating the purity of a monotone pattern *M* would be to make the numerator of the equation $M_{AB}$ + the maximum of $M_{A}$ and $M_{B}$, but that would mean that a pure block pattern was also classified as pure monotone (*M* = 1.0). Therefore, monotone purity includes an IIF condition that subtracts 1 from the numerator if none of the records are missing just one of the variables (see Equation 3).


$$\begin{array}{c} M=(M_{AB}+IIF(M_{A}+M_{B}=0,-1,max(M_{A},M_{B})))/(M_{AB}+M_{A}+M_{B}) \end{array}$$ (3)

### Visualization

Our method uses well-known but carefully chosen types of visualization (bar charts, line charts and heatmaps) to minimize the learning curve for users. The visualizations are built on top of Matplotlib and include keyword arguments (kwargs) that are passed through to that library so users may customize each plot in a variety of ways.

**Fig 3:**
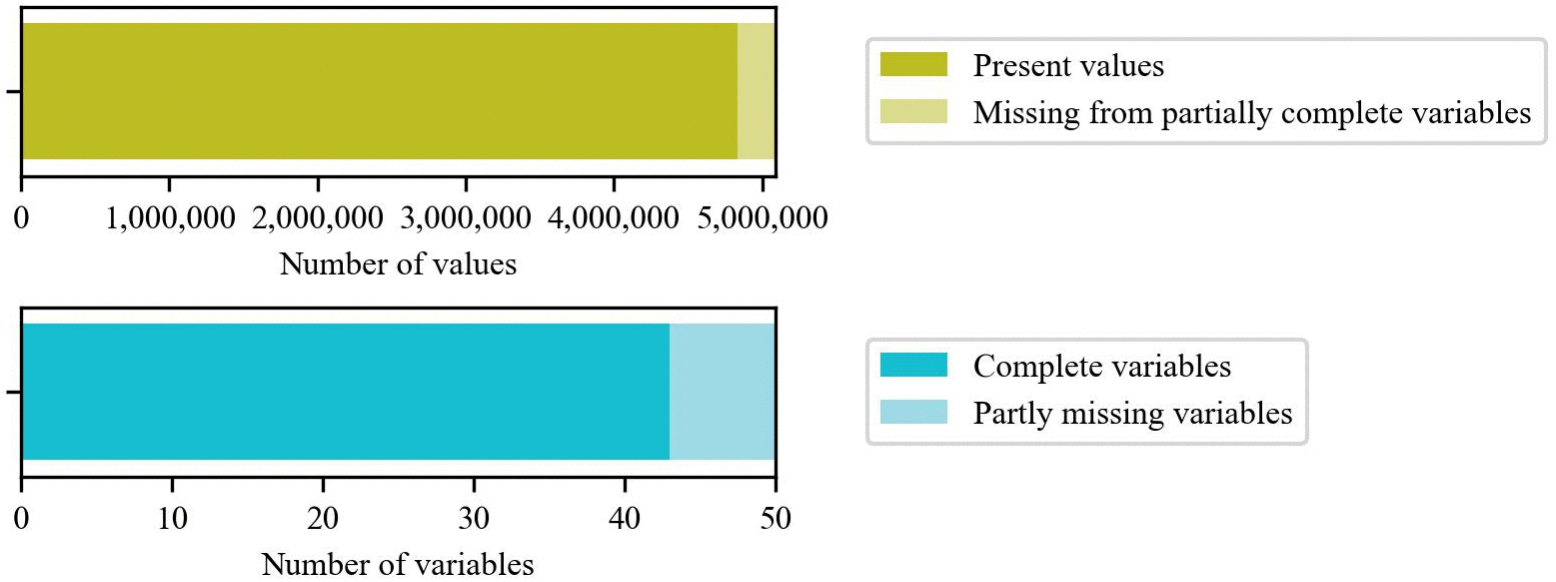
The dataset overview for the diabetes dataset [46].

**Dataset overview:** Two stacked bar charts provide an overview of the dataset’s missingness (Fig 3). In that example the variables are either complete or partly missing. If any variables were completely empty then they would have been labeled as *Missing from empty variables* and *Empty variables* in the two bar charts.

**Fig 4:**
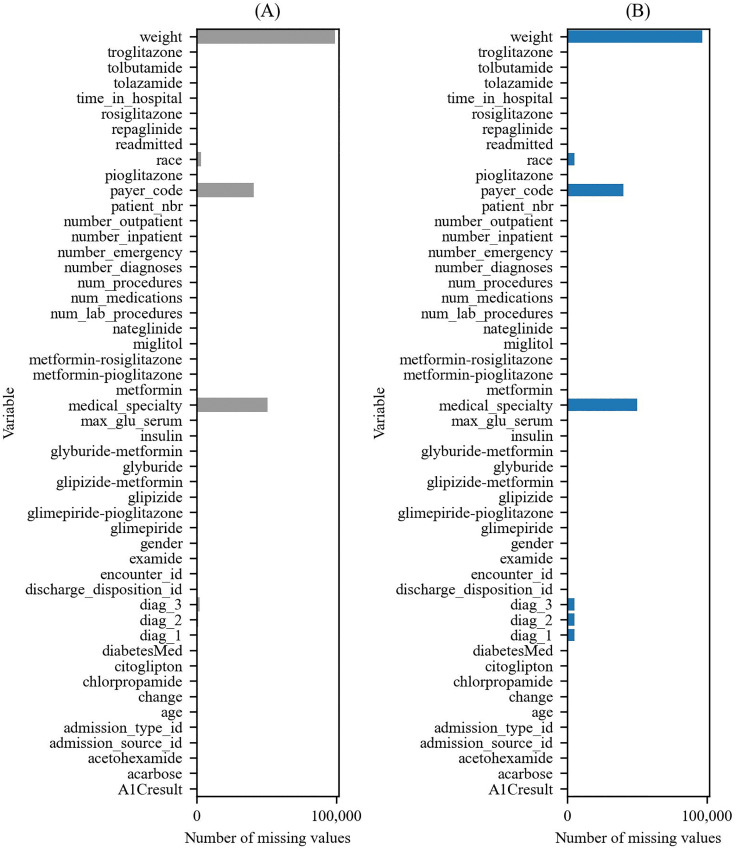
The variable overview for the diabetes dataset [46]. A normal bar chart (A) makes *diag_1* and *diag_2* appear to be complete, because there are too few missing values (21 and 358, respectively) for the bars to be visible. That is overcome in (B) because perceptual discontinuity has been applied to make all the non-zero bars long enough to be seen.

**Variable overview:** Datasets often contain some variables that are missing a few values and others that are missing many values. With a standard bar chart it may be difficult to distinguish complete variables from those that are missing a few values (Fig 4A). Therefore, we use perceptual discontinuity [31] to make sure that any variable with missing data stands out (Fig 4B).

**Fig 5:**
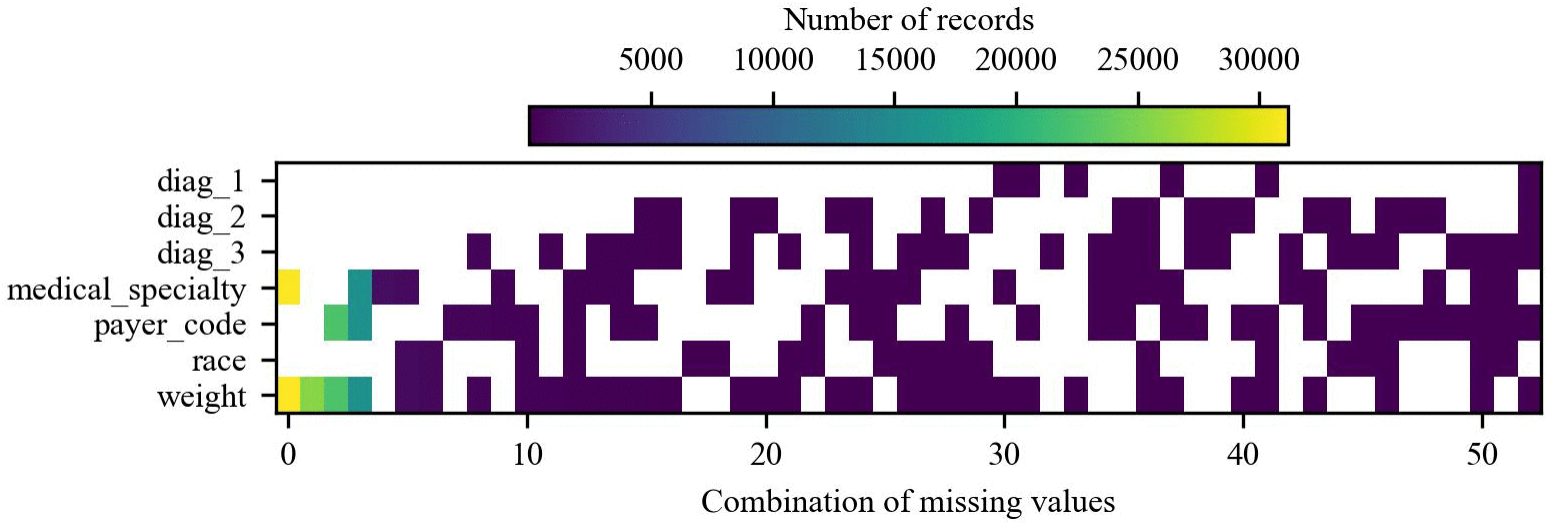
The 53 combinations of variables that are missing in the diabetes dataset [46]. The most common combination is *weight* and *medical_specialty*, which are missing together in 31,197 records.

**Combination heatmap:** It is also important to see which combinations of variables are missing together (Requirement **R2**). That is provided by a heatmap (Fig 5) which shows each combination of values that are missing together in a separate column, irrespective of how often that combination occurs (**R1**). However, the heatmap is not very suitable for highly multivariate datasets (**R3**) because the number of combinations may increase substantially with the number of variables.

We calculate the purity of each pattern (disjoint, block and monotone) for each pair of variables that are not complete. If *n* variables have missing values then there are *n(n-1)/2* pairs (i.e., the overall computational complexity is *O*(*n*^2^)). For each pair of variables, purity is calculated using the set intersection data structure, which is much faster than calculating purity directly from the columns of a raw dataframe (in the case studies, the diabetes dataset had 101,766 records but only 54 set intersections, and the hospital dataset had almost 21 million records but only 600,000 set intersections). For that hospital dataset, the purity pairs took an average of 0.08 seconds to compute on a 64GB Microsoft Azure virtual machine (3884 seconds for all 46,360 pairs). The vizdataquality package stores the purities in a dataframe that contains the pattern type (block, monotone or disjoint), purity and the two variables. The package also includes functions to export and import the purity dataframe, so they only have to be calculated once.

**Fig 6:**
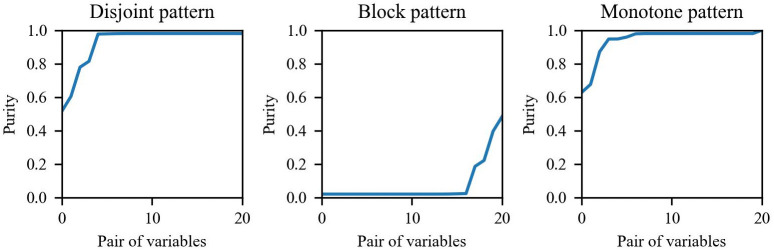
The disjoint, block and monotone pattern purity of each pair of incomplete variables in the diabetes dataset [46]. In each chart the pairs have been plotted in ascending purity order.

**Purity overview:** For this the purities are sorted into ascending order and plotted on line charts (Fig 6). Perceptual discontinuity is used to make sure that low purities are distinguishable from zero, and high purities are distinguishable from 1.0 (a completely pure pattern). Strengths of the purity overview are that it is agnostic to rarity of a pattern (Requirement **R1**) and is useful for highly multivariate datasets as well as those with few variables (**R3**). A limitation is that the overview only shows pairwise patterns (**R2**).

**Fig 7:**
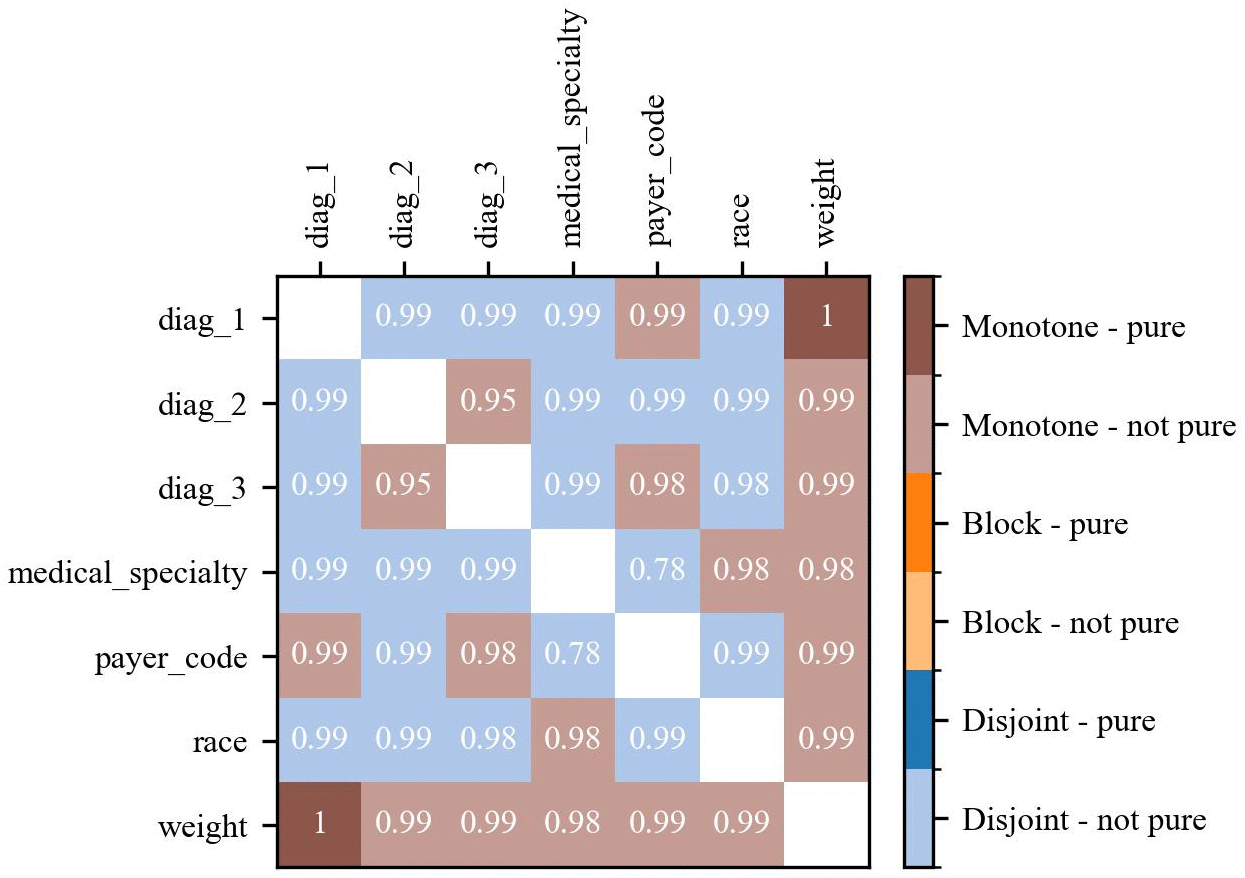
The most pure pattern for each pair of incomplete variables in the diabetes dataset [46]. The labels show each pair’s purity value.

**Purity heatmap:** To complement the purity overview we also provide a heatmap to let users see at a glance which pattern is most pure for each pair of variables. Hue is used to encode the type of pattern, with saturation distinguishing pure from impure pairs (Fig 7). The purity heatmap is also agnostic to rarity of a pattern (Requirement **R1**). At first glance, one might think that the heatmap is only effective for pairwise patterns but, as the NHS hospital admissions case study shows (see below), patterns involving many variables are also very clear (**R2**).

The purity heatmap function also has a threshold parameter, which enables users to filter the pairs (e.g., to show just those that are pure; threshold = 1.0) and simplify the heatmap. The purity threshold can reduce clutter to reveal patterns in highly multivariate datasets (Requirement **R3**). For example, as will be seen in the NHS hospital admissions case study, by setting the threshold to 1.0 to show only pure patterns the number of rows and columns was reduced from 389 (i.e., every variable) to 224 for the pure block pattern heatmap, and then down to 47 rows and columns in the pure disjoint pattern heatmap (NB: only one variable of each block was needed in that heatmap). The effectiveness of the method in that case study was helped by first investigating each type of pure pattern and then impure patterns.

### Explanation graph

Datasets often contain a variety of interwoven patterns of missing values. To help users iteratively investigate the patterns, functionality is provided for an *ExplanationGraph* that is made up of *ExplanationNodes*. A user creates each node by calling an *add_node()* function when they have identified a pattern that involves values that are missing from specific records, intersections and/or variables. Those missing values are then internally flagged as “explained”, and the corresponding variables and intersection IDs are stored with the node. Users may also provide a caption, description and attributes (shape, color, etc.) for the node. The explanation graph is, therefore, a tangible artifact that both documents the investigation and may help communicate the findings to stakeholders (Requirement **R5**).

The root node is zero and each time a user adds a node to the graph the node number is automatically incremented, capturing the order in which the user identified the patterns. When a node is created the graph automatically links it to existing nodes that contain any of the same variables and/or intersections. Nodes may also be added together, in which case there will not be links between those sibling nodes. Node positions may also be customized, to improve the automatically generated layout.

At any point in the investigation some missing values, variables, records and intersections are explained but others are not. If all of a variable’s missing values have been explained then the variable is termed “completely explained”, if some have been explained then the variable is “partly explained”, and if none have been explained then the variable is “not explained”. Similarly, at any given point in the investigation, each record and intersection is completely, partly or not explained.

**Fig 8:**
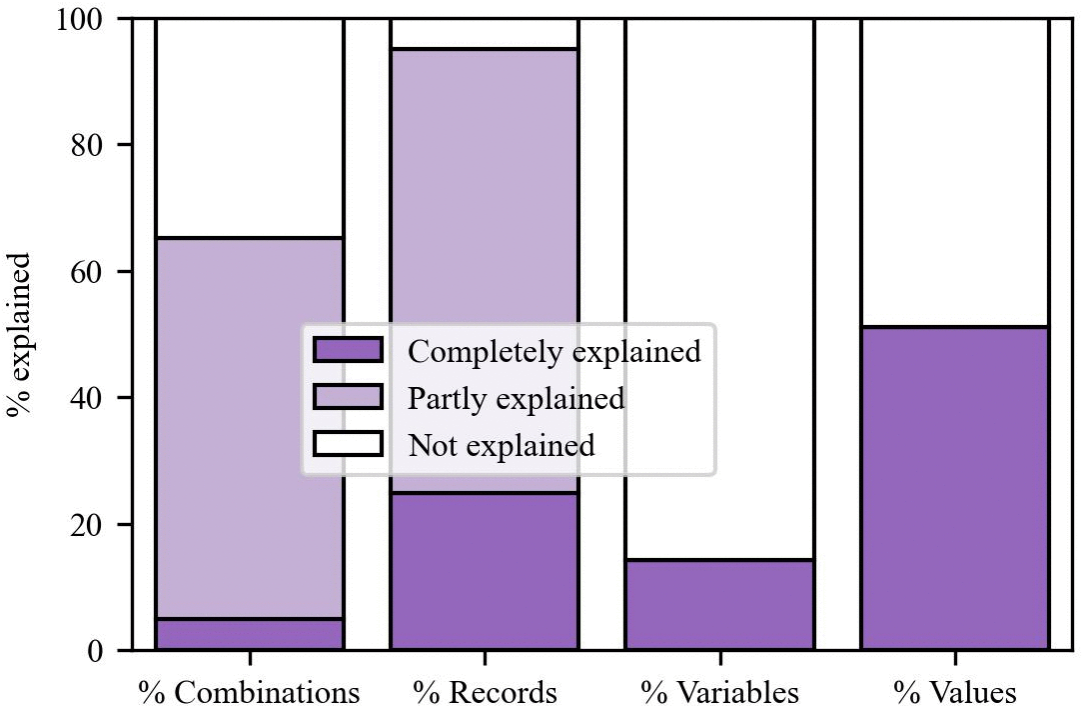
The percentage of missing values, records and combinations that are accounted for just by the weight variable in the diabetes dataset [46]. The partly and not explained values, records and combinations involve some of the other six variables that are sometimes missing.

**Explanation summary:** This stacked bar chart shows the percentage of missing values, variables, record and intersections that have been completely explained, partly explained or have not yet been explained (Fig 8). That helps to formalize the investigative process and is important for enabling users to keep track of the amount of work that remains to comprehensively account for all of the missingness at any point during the investigation (Requirement **R4**). Perceptual discontinuity is implemented to ensure that each bar is visible.

**Fig 9:**
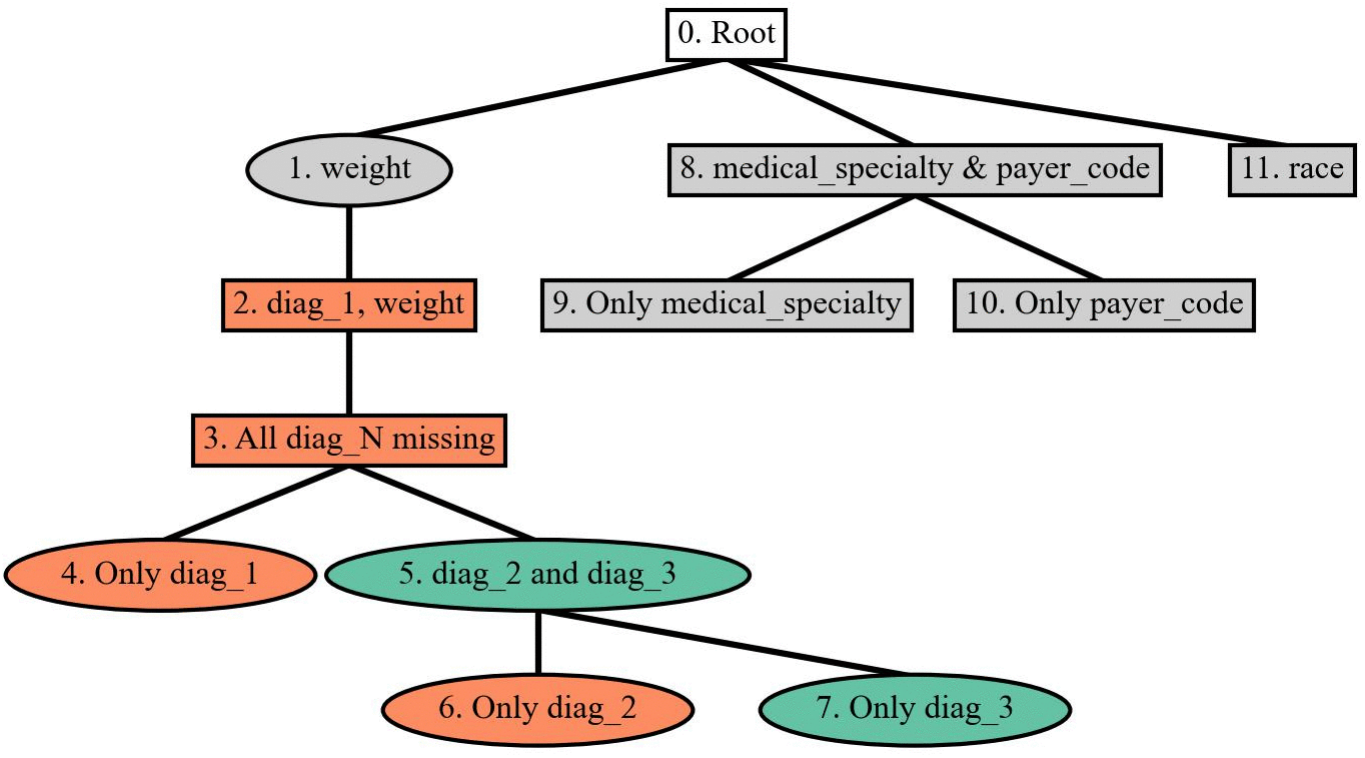
The final explanation graph for the diabetes dataset [46].

The vizdataquality package also includes functions to export and import the explanation graph, plot it (Fig 9) and output the details of every node to a table. These collective details aid reproducibility (Requirement **R5**), together of course with the Jupyter Notebook (or similar) that a user created.

### Workflow

Several example Jupyter notebooks are included with the vizdataquality package. One of those (*missing data structure 2.ipynb*) shows our recommended workflow, which users can of course copy and customize if required. The workflow steps are:

1. Compute the missingness data structures
2. Plot overview of missingness in dataset as a whole (Fig 3)
3. Plot the number of missing values for each variable (Fig 4b)
4. Visualize the combinations of missing values (Fig 5)
5. Calculate and visualize purities (Figs 6 and 7)
6. Iteratively:
  (a) Identify patterns
  (b) Investigate them to determine the underlying structures
  (c) Document the patterns/structures by adding them to an explanation graph
7. Check whether all missing values, variables, records and combinations have been explained (Fig 8)
8. Plot the final explanation graph (Fig 9)

## Case study: UCI diabetes dataset

This case study used the “Diabetes 130-US Hospitals for Years 1999-2008” dataset [46], which is freely available from the UCI Machine Learning Repository, to illustrate the visual analytic method. The dataset contains 101,766 records and 49 variables from 71,518 unique patients. The case study was performed on a Windows 11 laptop with 32GB RAM.

The dataset and variable overviews (Figs 3 and 4B) show that there are missing values in seven variables. The original paper [46] reported that only five variables had missing values. Two variables that it said were complete are in fact missing 358 (*diag_2*) and 21 values (*diag_1*). That error may have been caused by numerical rounding during preparation of the paper, but it should also be noted that those are the two variables that are almost invisible if perceptual discontinuity is not used (Fig 4A).

Almost every record is missing the *weight* variable, which may be explained by hospitals not being required to record weight before 2009 [46]. We make this univariate pattern the first node in the explanation graph. At this point, the explanation summary shows that *weight* accounts for approximately half of the values that are missing in the dataset, and about a quarter of the records are only missing that one variable (Fig 8). However, the summary’s “partly explained” bars show that about 70% of the records are missing a wide variety of combinations of other variables in addition to *weight*. A small number of records contain *weight* but are missing various combinations of the other six missing variables (see the “not explained” bars).

Purity calculations were performed using the block, disjoint and monotone metrics (see Equations 1–3). The purity overview shows that none of the pairs have a block pattern, but some have fairly pure disjoint and monotone patterns, and there is also a pure monotone pattern (Fig 6).

A purity heatmap shows that the pure monotone pattern is for *diag_1* and *weight* (Fig 7). Nine pairs have an almost pure monotone pattern (purity ≥ 0.98), 10 pairs have an impure disjoint pattern (purity ≥ 0.98), and *medical_speciality* and *payer_code* have a less pure disjoint pattern. That pure monotone pattern is probably not surprising given that *diag_1* is only missing in 0.02% of records whereas *weight* is missing in 97% of records. However, it is interesting to note that none of the other three variables with few missing values (*diag_2*, *diag_3* and *race*) exhibit a pure monotone pattern with *weight*.

Next we investigate the three diagnosis variables. The primary diagnosis *diag_1* is missing from 21 records. All but one of those contain values in *diag_2* and/or *diag_3* (the secondary diagnoses), which the purity heatmap flags as impure disjoint patterns (Fig 7). The reason for the impurity is because one record does not contain any diagnoses. Some records just contain a primary diagnosis (*diag_1*) or a primary and the first secondary diagnosis (*diag_1* and *diag_2*), which are patterns of missing data that are expected to occur in health data [21]. However, the additional secondary diagnosis (*diag_3*) should not be present if *diag_2* is missing.

As Fig 4 shows, approximately half of the *medical_specialty* and *payer_code* values were missing. Sometimes they were missing together but, as impure disjoint pattern in the purity heatmap indicates (Fig 7), those variables missing much more often in different records. Finally, *race* was missing in a small number of records.

The complete explanation graph is shown in Fig 9. The links indicate overlap between the variables in each pattern. Node 1 is the univariate *weight* pattern and node 2 involves two variables (*weight* and *diag_1*). Nodes 2 and 3 are linked because they both involve *diag_1*, and similar logic produces the subtree structure for nodes 4−7. Nodes 8−11 are in another subtree that is linked to the root because none of the variables (*medical_specialty; payer_code; race*) are involved in the other nodes’ patterns.

Using the user-definable node attributes, nodes 1, 3 and 5−7 have been drawn with ellipses because explanations are offered for those patterns. In health data it is correct that patient records may not contain secondary diagnoses, which is why nodes 5 and 7 are colored green. However, there should be a primary diagnosis which is why nodes 2−4 are colored orange. Also, if there is an additional secondary diagnosis (*diag_3*) then there should also be a secondary diagnosis (*diag_2*), which is why node 6 is colored orange.

Finally, to emphasize the insights that the new visual analytic method provides, Table 1 lists the missingness correlations between variables that were not complete. Most of the correlations are close to zero, which is uninformative. That includes the *medical_specialty x payer_code* correlation, which is actually a disjoint pattern (Fig 7) because those variables are missing separately (56% of records) more often than they are missing together. Only the correlation for *diag_2* and *diag_3* stands out, and the value (0.387) suggests that they may be in a block pattern. There are two ways in which that is misleading because and as noted above: (a) they actually have an impure monotone pattern (Fig 7; both missing in 279 records; only *diag_3* missing in another 1144 records), and (b) the impurity is caused by a data quality issue (*diag_2* is missing by itself in another 79 records).

**Table 1:** Pearson correlation coefficients of missingness for the diabetes dataset [46] variables that were not complete. Missingness correlations ar calculated by setting missing and present values equal to zero and one, respectively [25].

Variable A	Variable B	Correlation
medical_specialty	payer_code	−0.137
payer_code	race	−0.048
diag_3	medical_specialty	−0.045
diag_2	medical_specialty	−0.026
race	weight	−0.025
diag_1	medical_specialty	−0.003
diag_2	weight	−0.001
medical_specialty	weight	0.002
diag_1	weight	0.003
diag_1	payer_code	0.004
diag_1	diag_3	0.004
diag_2	payer_code	0.007
diag_1	race	0.007
medical_specialty	race	0.009
diag_3	weight	0.010
diag_1	diag_2	0.011
diag_2	race	0.016
diag_3	race	0.024
diag_3	payer_code	0.038
payer_code	weight	0.044
diag_2	diag_3	0.387

## Case study: NHS hospital admissions dataset

This second, much more challenging case study used a dataset that contained a whole year (2015–16) of pseudonymized Admitted Patient Care (APC) health records for 564 hospitals in England. In APC data each record is for a unique episode of treatment that takes place after a patient has been admitted to hospital. The record contains demographic, diagnosis, treatment and other data. APC data is used by clinicians, to pay hospitals, for reporting care quality and for a wide variety of research [47].

APC data must be held in a Trusted Research Environment (TRE) that has NHS Data Security and Protection Toolkit accreditation. We used Microsoft Azure Windows 10 virtual machines with 16GB and 64GB RAM in the University’s LASER TRE.

The dataset contained 20,724,064 records for 1,655,846 unique patients. That makes the dataset one of the largest ever used in research developing new visual analytic techniques [48].

The APC dataset originally had 567 variables, but 178 were completely empty so were removed before starting this case study. A 314 page data dictionary documents each variable, including any special values that are used to indicate null values (e.g., 1800-01-01 for some dates) [49]. The dataset was pre-processed to set those special values to *np.nan* so they would be treated as null by the Python Pandas package.

We followed the workflow described above. Step 1 showed that there were 595,171 combinations of missing values (an incredibly large number for set-based data), which at one extreme occurred in just one record and at the other occurred in 103,046 records. The intersections involved 49–286 variables, indicating that the missingness patterns were very interwoven.

**Fig 10:**
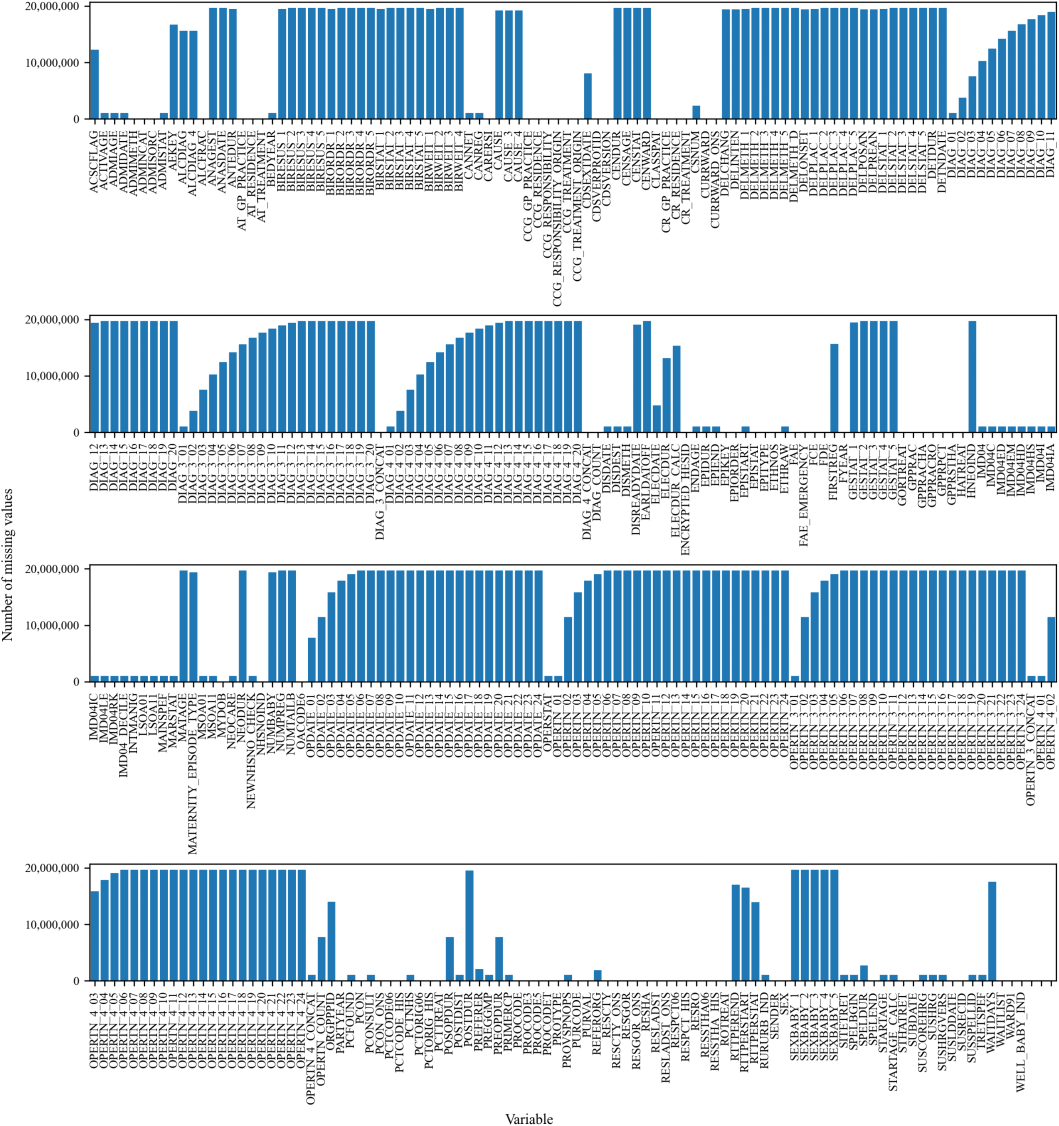
Variable overview showing the number of missing values in each variable of the APC dataset.

The variable overview (Fig 10) suggested that seven groups of variables had an increasing number of missing values (e.g., DIAG_01 to DIAG_20), which would be expected (DIAG_01 is the primary diagnosis and the others are secondary). However, it was impossible to tell whether those variables formed a monotone pattern because each pixel in the bars represented 100,000 records (that is a fundamental limitation of a bar chart’s ability to discriminate between values). Most of the other variables appeared to be either almost complete or missing most values.

**Fig 11:**
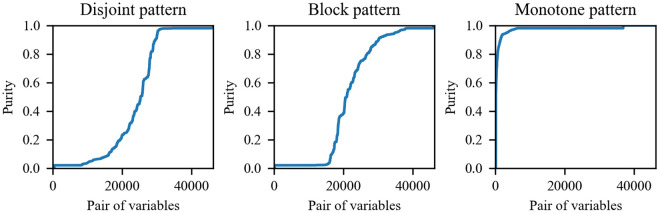
The disjoint, block and monotone pattern purity of each pair of incomplete variables in the APC dataset.

Purity calculations, performed using Equations 1–3, showed that about 20% of the pairs of variables had a pure monotone pattern of missingness (Fig 11). Even though fewer of the other patterns were pure, there were still 425 pure block pairs and 146 pure disjoint pairs.

**Fig 12:**
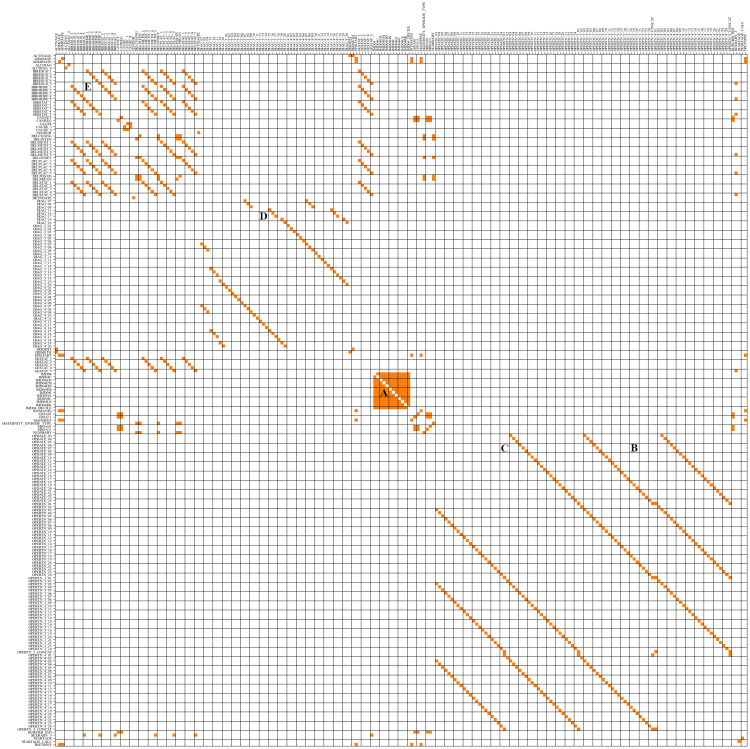
The 224 variables that had pure block patterns with one or more other variables. The annotated patterns are: (A) deprivation variables (all beginning IMD), (B) operation codes (OPERTN_01, etc.), (C) operation dates and codes (OPDATE_01, OPERTN_01, etc.), (D) diagnosis codes (DIAG_07, etc.) and (E) birth variables (BIRORDR_1, etc.).

**Fig 13:**
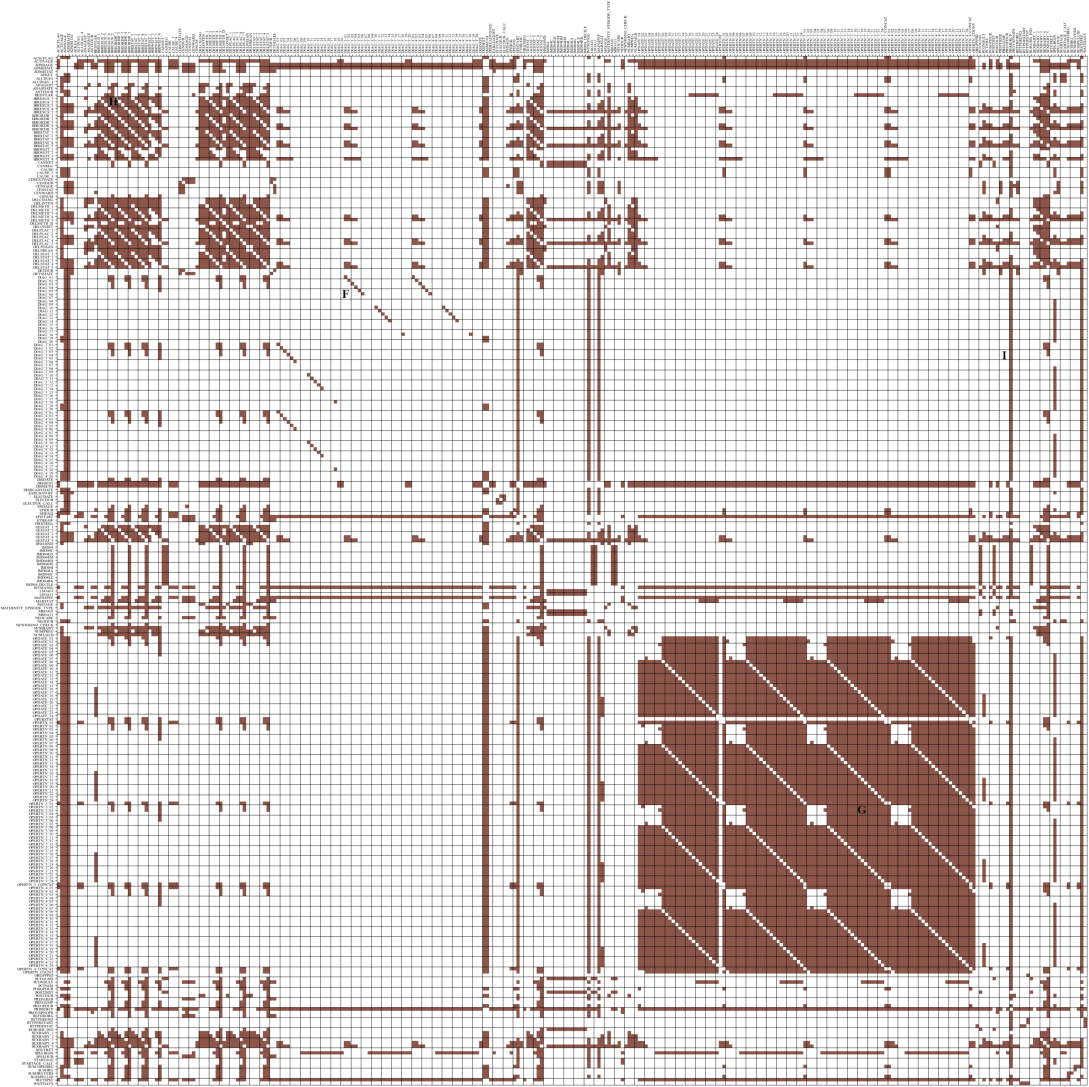
The 305 variables that had pure monotone patterns with one or more other variables. The annotated patterns are: (F) diagnosis codes (DIAG_3_01, etc.), (G) operation dates and codes (OPDATE_01, OPERTN_01, etc.), (H) birth variables (BIRORDR_1, BIRWEIT_1, SEXBABY_1, etc.), and (I) PRIMERCP.

**Fig 14:**
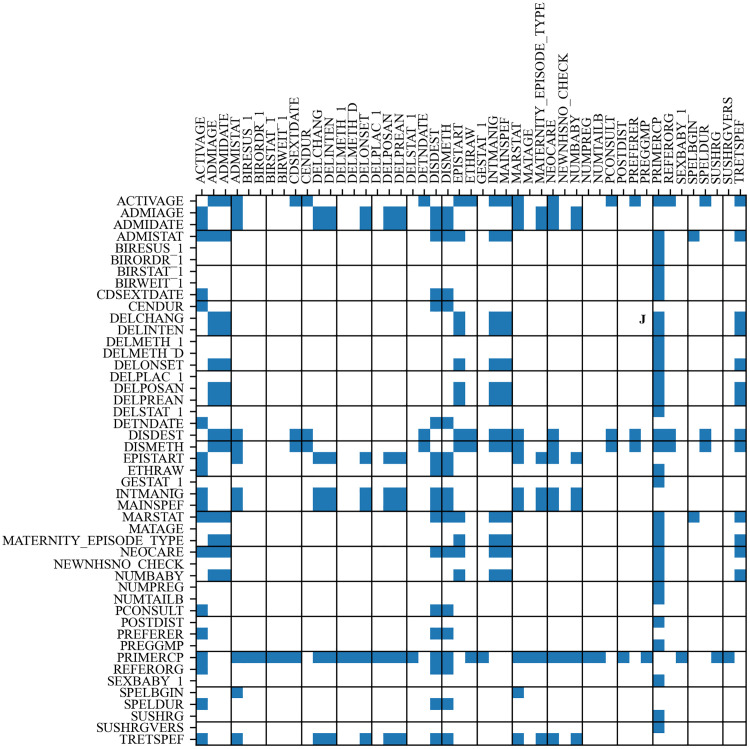
The 25 block variables and 22 other variables that had pure disjoint patterns with one or more other variables. The annotated pattern is: (J) PRIMERCP.

Selecting only the pure pairs (purity = 1.0) and visualizing them with heatmaps (Figs 12, 13 and 14) was much more informative than the bar charts, and provided some breakthrough insights. The first was PRIMERCP (an organization code), which popped out because it had a pure monotone or disjoint pattern with every other incomplete variable (Figs 13I and 14J), so we made it Node 1 in the explanation graph. In fact PRIMERCP was only missing one value, which it is reasonable to assume was caused by a rare data quality issue.

**Fig 15:**
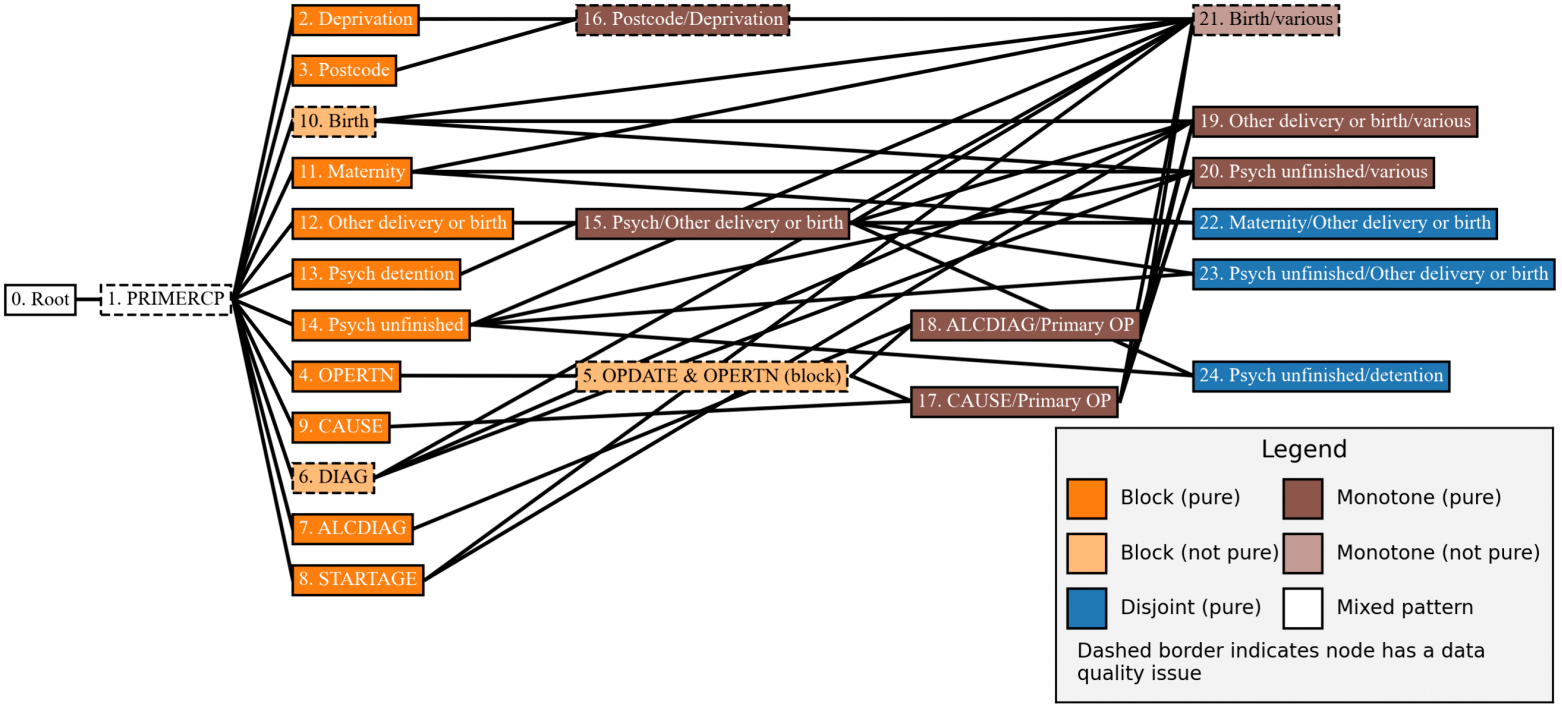
An explanation graph showing the block and inter-block patterns for the APC dataset. Nodes 0−14 are the same as those in Fig 16, but the other nodes are different.

**Fig 16:**
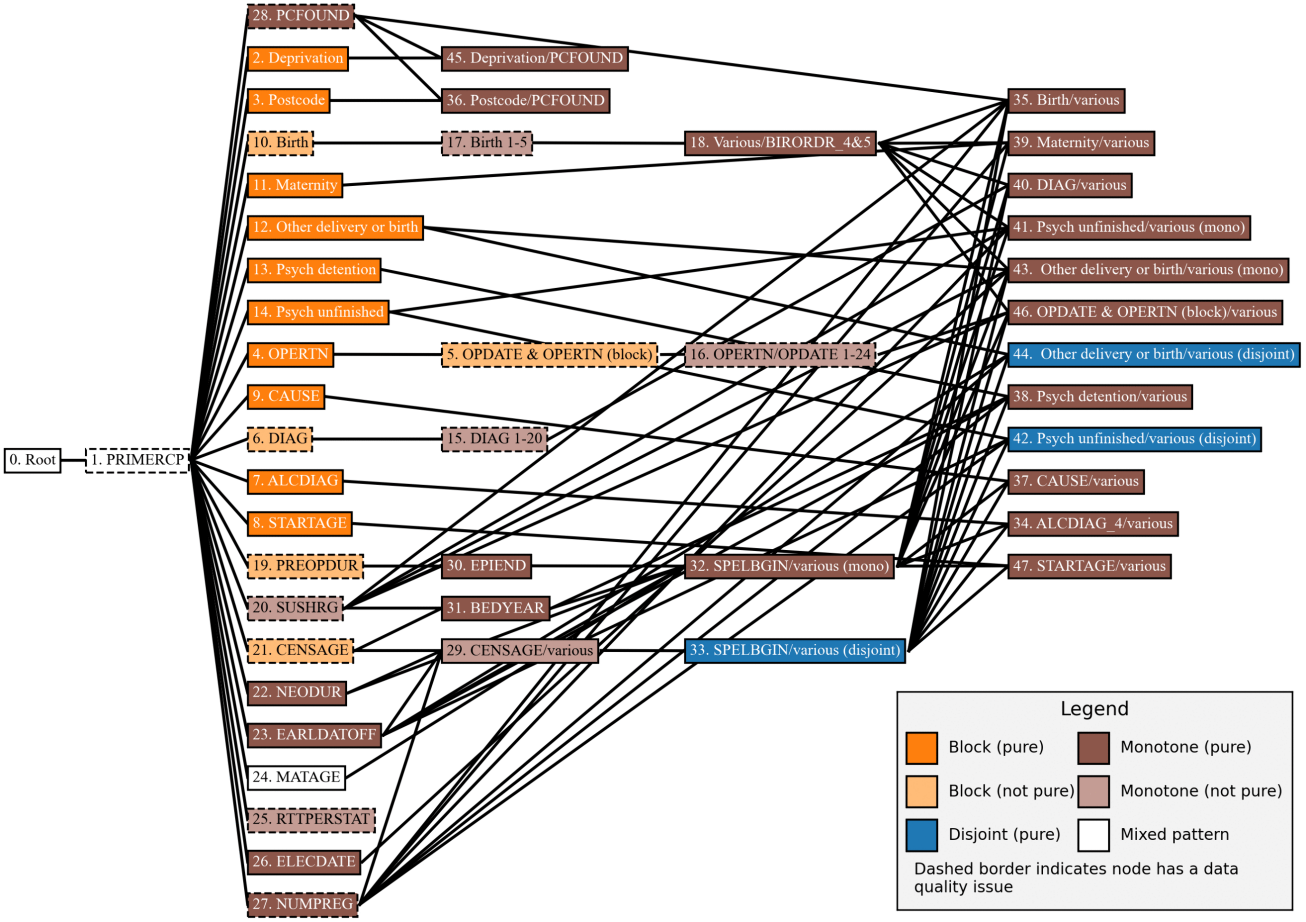
An explanation graph showing the patterns for non-block variables for the APC dataset. Nodes 0−14 are the same as those in Fig 15, but the other nodes are different.

For the iterative part of the workflow (Step 6) we investigated block patterns and then patterns involving non-block variables. Originally we had intended to show all of the patterns/structures in a single explanation graph, but the network became too cluttered. Therefore, it was divided into two graphs (Figs 15 and 16) in which nodes 0−14 are the same, but the other nodes are different.

### Block patterns

The pure block heatmap contained 224 rows and columns, with several eye-catching diagonal lines (Fig 12). The variables were ordered alphabetically in the heatmap, which helped patterns involving similarly named variables to stand out. The patterns were investigated by iteratively identifying blocks, and then filtering them out to further simplify the heatmap so any remaining patterns became more salient. The filtering was straightforward to achieve by writing a purity filtering function with a “names” parameter to include/exclude records that involved the named variables or any variables with one of the names as a stem. That type of on-the-fly computation is a hallmark of visual analytics.

#### Derived variables.

In the explanation graphs (Figs 15 and 16) nodes 2−9 involve derived variables. One such block was for 12 deprivation variables (all beginning IMD; Fig 12A), which are socio-economic measures derived from the region where a patient lives. Seven other variables (CANNET, etc.) are all derived from a patient’s postcode and were also in a pure block.

The diagonal lines in the heatmap were for groups of variables that are derived from each other. In APC data 24 variables store codes for a patient’s operations (OPERTN_01 is the primary procedure, and OPERTN_02 to OPERTN_24 are for secondary procedures) and 48 other variables (OPERTN_3_01, OPERTN_4_01, etc.) contain three- and four-character versions of those codes. Those 24 blocks (Fig 12B) were combined into a single explanation graph node (OPERTN).

There were also 24 variables for the dates of the procedures. For every procedure there should be a corresponding date (Fig 12C), making a pure block pattern, but that was not the case for OPDATE_01 and OPDATE_02 and detailed analysis uncovered three reasons. First, in one record the dates were missing but the procedure codes were present. Second, in 7.7 million records OPERTN_01 was ‘-’, which the data dictionary states is the code for “No operation performed” and therefore explains the missing dates in those records. Third, in the same 7.7 million records the value of OPERTN_4_01 was padded out to four characters (“- ”). That conflicted with the data dictionary, which stated that the value should be ‘-’ (i.e., not padded). That type of error can easily cause analysts to develop data processing code that does not work correctly.

There are also 20 variables for a patient’s diagnoses (DIAG_01, etc.) and 40 more variables contain three- and four-character versions of those codes (DIAG__3_01, DIAG_4_01, etc.). All of the three- and four-character variables were in block patterns (Fig 12D), which again appeared as diagonal lines of heatmap cells. However, 12 of the full diagnosis code variables were not part of those patterns, and the reasons appear to be spurious extra characters at the end of some values and a typo in the definition of a special value in the data dictionary (R96X (the code for sudden death) rather than R69X (illness, unspecified)).

There were pure block patterns for two alcohol-related diagnosis variables (ALCDIAG; ALCDIAG_4), STARTAGE and STARTAGE_CALC which store a patient’s age as an integer and decimalized (to cater for babies), and three injury or poisoning diagnosis variables (all beginning CAUSE).

#### Associated variables.

Some variables store different information, but are in pure blocks and so must be associated with each other. They are shown as nodes 10−14 in the explanation graphs (Figs 15 and 16).

To accommodate multiple births (twins, etc.), APC data has groups of variables whose names end with a digit. Those groups (BIRORDR_1 to BIRORDR_5) were shown as parallel diagonal lines of heatmap cells (Fig 12E), which were missing SEXBABY for births 1–4, and were combined into one node (Birth).

NUMBABY and six other variables that contain information about a mother’s maternity record were missing in a pure block pattern. In other words, as expected, those variables were only present in the 1.3 million records that were for maternity episodes.

Another 9241 record pure block involved six variables that store episode/admission dates, a patient’s age, the plan for the patient and the consultant’s specialty (EPISTART, etc.). They were only missing for “other” delivery and birth events (EPITYPE = 5 or 6).

CENDUR (duration of care for psychiatric patients) and DETNDATE (a date for patients under a detention order) were in a pure block pattern. ACTIVAGE, DISDEST and DISMETH were also all missing in a pure block pattern, in 3175 records. Further analysis showed that this block were the records for unfinished episodes (EPISTAT = 1) of psychiatric patients (EPITYPE = 4).

People who are experts in the coding of NHS data might be familiar with the above associations, but that is not the case for most analysts or researchers. The absence of such knowledge would be a barrier to them, so a clear benefit of our workflow is the ability to make such associations explicit.

### Inter-block patterns

Next we investigated pure monotone or disjoint patterns that existed between the above blocks. That was straightforward to achieve by applying the purity filtering function to only include records about one of each block’s variables, and using the function again to output the inter-block patterns. That investigation revealed several monotone patterns (nodes 15−21 in Fig 15), including:

- Detained psychiatric patients always had values for the episode start date (EPISTART) and the other five variables in that block.
- The deprivation variables were missing in 100,000 more records than the postcode variables, perhaps because some patients did not live in England.
- There was always a primary procedure (OPERTN_01) for patients with an injury or poisoning diagnosis (CAUSE) or an alcohol-related diagnosis (ALCDIAG).
- The other delivery and birth events block (EPISTART, etc.) had pure monotone patterns with the STARTAGE block (which the data dictionary states should be null for other maternity events), the diagnoses blocks (DIAG_01 to 20) and several other blocks.
- The unfinished psychiatric records block (DISDEST) had pure monotone patterns with several blocks, including the DIAG_19 and DIAG_20 blocks which indicated that those psychiatric patients sometimes had complex health conditions (the other 18 diagnosis codes were used).
- The five birth variable blocks (BIRORDR_1, etc.) formed a variety of pure and impure monotone patterns with other blocks, which highlight that analysis of births could be affected by data quality issues. For example, there was no deprivation value for 8% of births, and 23,000 records were missing the baby’s STARTAGE.

Three inter-block pure disjoint patterns also occurred (nodes 22−24 in Fig 15). One showed that unfinished psychiatric records never involved other delivery and birth events, but the other two were less obvious:

- Maternity records never contained other delivery and birth events.
- Unfinished psychiatric records were never for detained psychiatric patients.

### Patterns involving non-block variables

The next stage of the analysis was to investigate patterns between the 81 variables that were not involved in any block patterns. This involved the following steps.

#### Monotone heatmap.

The first step derived insights from the patterns that are shown in the monotone heatmap (Fig 13). That resulted in the nodes 15−17 in Fig 16.

APC data contains 20 variables for a patient’s diagnoses, which should be filled up in ascending order (e.g., DIAG_01 then DIAG_02, etc.), and only patients with the most complex health conditions/treatment would have a complete set. That should exhibit itself as a pure monotone pattern for the DIAG variables, so the almost total absence of that pattern is striking (Fig 13F). Further analysis showed that the cause was gaps in 7219 records for the DIAG variables, with 3712 being for accident and emergency or dental casualty department admissions.

Similarly, there are 24 variables for a patient’s operations and corresponding dates so the hole for some pairs of OPERATN and OPDATE was striking (Fig 13G). Further analysis showed that 314 records contained gaps in the OPERTN and OPDATE variables, with 302 of the records being for a single admission method (admitted ante-partum) and 294 from one health provider.

As explained above, APC data has groups of variables for births, so the pure monotone pattern for BIRTHORDR_1 to 5 was expected (e.g., the first two birth groups were always used if there were twins; Fig 13H). Each group should exhibit a block pattern, but a heatmap showed that some were actually in monotone patterns, and further analysis showed that BIRWEIT and SEXBABY were missing for some births. In fact SEXBABY_1 was a common data quality issue, because it was missing in 674,764 records that had a BIRORDR_1 value, and on 99.98% of those occasions it was a single birth.

#### Hybrid heatmap.

**Fig 17:**
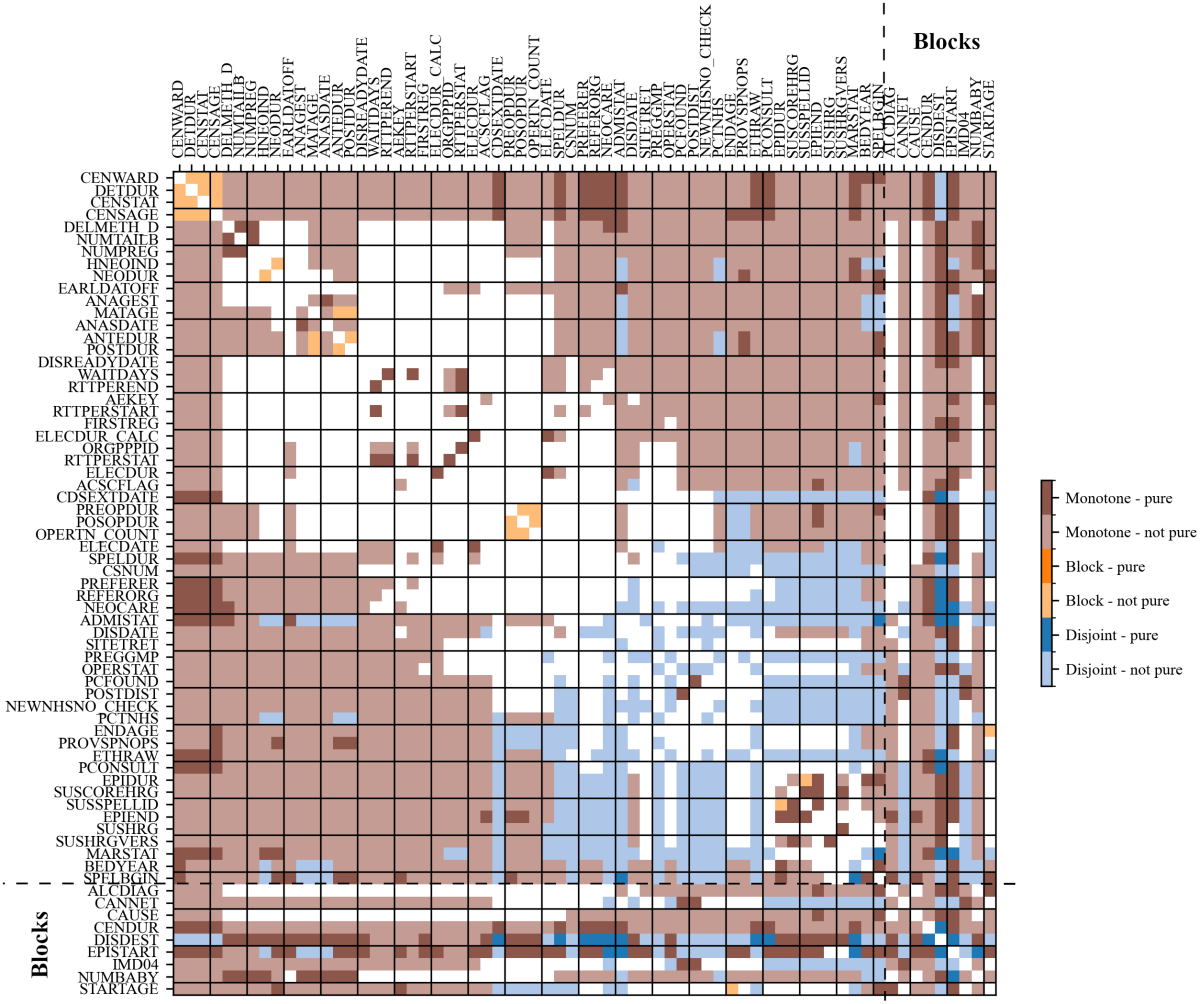
The hybrid heatmap, with one variable for each of the nine blocks that are involved are listed alphabetically in the righthand columns and bottom rows (see also Table 2). The other rows/columns are for 58 non-block variables and are listed in descending order of number of missing values, from CENWARD (20,721,474 missing) to SPELBGIN (9253 missing).

**Fig 18:**
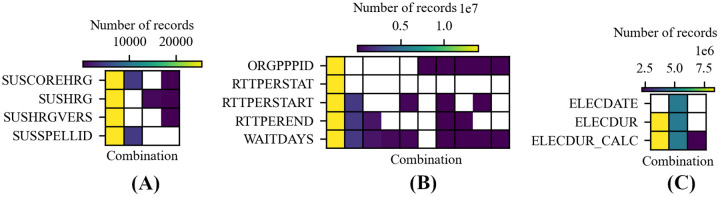
Combination heatmaps showing the number of records that contained each combination of missing values for: (A) Four system variables, (B) five referral variables, and (C) three elective admissions variables.

At this point 58 variables were still unexplained so, after some dead-end investigations, we took a more holistic approach and visualized them in a heatmap that showed monotone, block and disjoint patterns with purity ≥ 0.999 in the context of the pure blocks (Fig 17). The patterns that correspond to nodes 18−33 in Fig 16 only involved non-block variables and included:

- The impure block for PREOPDUR, POSOPDUR and OPERTN_COUNT was probably due to a data quality issue because all three values were present in 7.8 million records, but another 26,294 records only had one or two of the values.
- Four system variables (SUSCOREHRG, SUSHRG, SUSHRGVERS, SUSSPELLID) were all present in the vast majority of records (99.85%). In 25,535 of the other records those variables were all missing together, but in the remaining 4902 records only one or two of the variables were missing (Fig 18A). That appears to be a data quality issue because the data dictionary gives the same null criteria for the three of the variables, but indicates that the fourth (SUSHRGVERS) should never be missing.
- Five referral variables (RTTPERSTAT, RTTPERSTART, RTTPEREND, ORGPPPID, WAITDAYS) had a complex pattern. The four most frequently occurring combinations of missing values obeyed a monotone pattern, but the other four combinations revealed two data quality issues (Fig 18B). One issue was that the referral start date (RTTPERSTART) was missing in 185,326 records that had an end date (RTTPEREND). The other issue was that some referrals appeared to be part of a designated patient pathway (ORGPPPID) but others did not.
- Three elective admissions variables (ELECDATE, ELECDUR, ELECDUR_CALC) adhered to a pure monotone pattern, with all three variables missing in some records, other records missing ELECDUR and ELECDUR_CALC, and others just missing ELECDUR_CALC (Fig 18C). That pattern appears to be correct because the data dictionary states that elective admission dates can be in the future, ELECDUR can only be calculated after a patient has been admitted, and ELECDUR_CALC is only used for a subset of admissions.
- Three maternity variables (NUMPREG, DELMETH_D, NUMTAILB) had a pure monotone pattern with each other. That appears to be a data quality issue, because NUMPREG (the number of previous pregnancies) is missing less often than the other variables, and the data dictionary states that DELMETH_D (the delivery method) should be ‘X’ if it is not known and NUMTAILB (the number of valid baby groups) should default to ‘1’.
- PCFOUND (postcode found) and POSTDIST (the first part of a postcode) had a pure monotone pattern, because 10,472 of the POSTDIST values were not valid postcodes.
- SPELBGIN had pure monotone patterns because if it was missing (“not applicable” according to the data dictionary) then so were nine other variables. However, pure disjoint patterns showed that information about psychiatric patients was always present if SPELBGIN was missing.

**Table 2:** The nine block variables shown in Fig 17 and the corresponding nodes in Fig 15 and Fig 16.

Block variable	Corresponding node
ALCDIAG	7. ALCDIAG
CANNET	3. Postcode
CAUSE	9. CAUSE
CENDUR	13. Psych detention
DISDEST	14. Psych unfinished
EPISTART	12. Other delivery or birth
IMD04	2. Deprivation
NUMBABY	11. Maternity
STARTAGE	8. STARTAGE

Finally, nodes 34−47 in Fig 16 represent pure patterns between block and non-block variables. The many links between those nodes and the others in the graph may be grouped into the following generic explanations:

- Non-block variables missing 100+ times more values than block variables (e.g., NUMPREG and DISDEST, indicating that delivery records were independent of unfinished psychiatric records).
- Block variables missing 100+ times more values than non-block variables (e.g., CAUSE and SPELBGIN, indicating that the beginning of spell indicator was always present for patients with an injury or poisoning diagnosis.
- The number of missing values in a pair of block and non-block variables differed by less than 10% (e.g., EPISTART and SPELBGIN, where the extra EPISTART values were all 1801-01-01 which indicates an invalid date was entered).
- An intermediate difference between a pair of variables (e.g., EPISTART and EPIDUR, where 20,287 of the extra EPIDUR values were for unfinished episodes (EPISTAT = 1) but the other 486 extra records were unexplained).

## Discussion

This case study used the visual analytic method to investigate the patterns and structures of missing data in a large dataset (21 million records; 389 variables). ESI calculations detected 595,171 ESIs (each ESI was a unique combination of missing variables), which were simplified into 57 multivariate patterns that involved from 2 to 305 variables and accounted for all 4.5 billion of the missing values. For 35 of the patterns the results described above include explanations about why a pattern occurred, in terms of information from the data dictionary or attributes of the data (i.e., a structure of missingness rather than just a pattern). The majority of the pure patterns are accompanied by explanations about why it is correct that data is missing, but 14 impure patterns appear to involve data quality issues.

The very large number of ESIs was produced by the interwoven nature of many of the patterns (50% of the ESIs involved at least 202 variables). It would have been impossible to visualize them all directly, but filtering the purity heatmaps by pattern type and threshold proved to be a very capable way of producing insights for users. For example, the pure purity heatmaps (Figs 12-15) showed a variety of patterns that it was correct for the data to have, as well as revealing other patterns that were caused by a variety of data quality issues. Additionally, the hybrid heatmap (Fig 17) revealed many other patterns from its spatial and color clusters. When a specific pattern is expected to occur, it would be possible to check by calculating ESIs for that subset of variables and visualizing them in combination heatmaps or upset plots (e.g., the pure block OPERTN pattern or impure monotone DIAG 1–20 pattern; nodes 4 and 15, respectively, in Fig 16). However, having taken that approach in previous work [21,26], experience shows that the purity heatmaps made it faster to comprehensively investigate the patterns of missingness and far less reliant than a priori knowledge about the dataset.

The explanation graph documents the investigation and provides a visual overview that will help analysts put the many patterns into context and aid communication with stakeholders. Some general insights can also be drawn from the explanation graph. First, all but one of the pure and impure blocks appear at the same level of the graph, which shows that they involve different variables to each other. The exception is node 5, which combines operation codes with dates. Second, seven of the monotone patterns and a mixed pattern (node 24) are independent of the block variables, as shown by the presence of those patterns in the graph level that also contains all but one block. Third, disjoint patterns are less common than monotone patterns.

## Conclusions

A grand challenge in missing data concerns exploring the geometry of structured missingness [5]. In this research we developed a novel visual analytic method to enable users to investigate patterns of missing data and understand the underlying structures, addressing five core requirements. Two case studies were used to demonstrate the method’s utility and unique capabilities. One case study used a freely available dataset from the UCI Machine Learning Repository [46] and the other case study used a 21 million record/389 variable national dataset about patients admitted to hospitals.

The method is based on three patterns (block, monotone and disjoint) and includes new metrics that distinguish between those patterns and quantifies whether they are pure or impure. Bar and line charts are used to provide users with dataset, variable and purity overviews, and heatmaps show purity patterns and combinations of variables that are missing together.

The method’s capability for revealing rare patterns (Requirement **R1**) was demonstrated in two ways. First, a bar chart with perceptual discontinuity showed that two variables that the original UCI paper reported as complete [46] were actually missing small numbers of values (0.02% and 0.3%, respectively). Second, the purity metrics and heatmaps revealed a rare data quality issue (0.08% of records) involving the diagnosis variables, which was not apparent at all when correlations [25] were used to analyze patterns between those variables. The purity metrics and heatmaps also revealed a variety of rare patterns in the NHS case study.

One way of showing patterns that involve any number of variables (Requirement **R2**) is to visualize ESIs directly in an upset plot (e.g., [35]) or a combination heatmap (e.g., [37]). However, even with a modest number of combinations the patterns tend to become noisy (e.g., see Fig 5). A different way is to use our new purity heatmaps iteratively to show the three core patterns (block, monotone and disjoint) at user-definable purity thresholds. The NHS case study demonstrated the utility of those purity heatmaps for highly multivariate datasets (**R3**), reducing the 595,171 ESIs to a much smaller number of comprehensible patterns that popped out to users. Explanations of those patterns included relationships between blocks of missingness and the values of other variables, both common and rare data quality issues (e.g., the sex of babies often not being recorded or gaps in sets of diagnoses), and errors in the data dictionary.

The method’s explanation graph captures the order in which a user identified the patterns and relationships between them, which should help the findings to be communicated and aids reproducibility (Requirement **R5**). By visualizing the percentage of values, variables, records and missingness combinations that are included in the graph at any stage during analysis the explanation summary is invaluable for helping users ensure that they account for all of the missingness in a dataset (**R4**). To our knowledge, no other tools provide a capability like the explanation graph and summary.

One limitation is that the method is designed for data that is stored in a tabular format. That includes single data tables (like the two case studies) and relational data stored as tables. Time-series data is often stored in tables and so is handled to some extent, but the method does not include specific time-series functionality such as detecting time gaps. The method has not been designed for hierarchical or graph-structured datasets. A limitation of the case studies is that the explanations about the missingness patterns were not independently verified by domain experts. Many of the hospital case study patterns were explained using the data dictionary [49], which documents every variable and is particularly useful for users who are not trained as clinical coders (e.g., health analysts, epidemiologists and other researchers). With input from domain experts, some of those explanations may have been modified and gaps filled for the unexplained patterns. An inference capability about missing data mechanisms (MCAR, MAR and MNAR [4]) could be added to the method to inform those explanations.

Three areas for future work stand out. The first is to investigate different methods for laying out the explanation graphs, incorporating techniques such as edge bundling to improve legibility [50], and making the graphs interactive so users could highlight nodes that are linked to each other or involve specific variables. Second is a comprehensive comparison of our method to other approaches, including AI-based approaches that will no doubt be developed in coming years. Finally, we plan to build a catalog of exemplars by helping other researchers apply the method to new datasets.

## References

[1] Amusa LB, Hossana T. An empirical comparison of some missing data treatments in PLS-SEM. PLoS One. 2024;19(1):e0297037. doi: 10.1371/journal.pone.0297037 38241223 PMC10798466

[2] Hoevenaar-Blom MP, Guillemont J, Ngandu T, Beishuizen CRL, Coley N, Moll van Charante EP, et al. Improving data sharing in research with context-free encoded missing data. PLoS One. 2017;12(9):e0182362. doi: 10.1371/journal.pone.0182362 28898245 PMC5595279

[3] Templ M, Kowarik A, Alfons A, Prantner B. Visualization and imputation of missing values. 2019.

[4] Rubin DB. Inference and missing data. Biometrika. 1976;63(3):581–92. doi: 10.1093/biomet/63.3.581

[5] Mitra R, McGough SF, Chakraborti T, Holmes C, Copping R, Hagenbuch N, et al. Learning from data with structured missingness. Nat Mach Intell. 2023;5(1):13–23. doi: 10.1038/s42256-022-00596-z

[6] Ruddle RA, Hama L. vizdataquality 1.1.0. 2024. Available from: https://pypi.org/project/vizdataquality/

[7] Ruddle RA, Cheshire J, Fernstad SJF. A practical guide to characterising data and investigating data quality.

[8] King T, Schwarzenbach J. Managing data quality: A practical guide. BCS Publishing; 2020.

[9] Alemzadeh S, Niemann U, Ittermann T, Völzke H, Schneider D, Spiliopoulou M, et al. Visual analysis of missing values in longitudinal cohort study data. In: Computer Graphics Forum. vol. 39. Wiley Online Library; 2020. pp. 63–75.

[10] Deva C, Dixon L, Urban M, Ramirez‐Villegas J, Droutsas I, Challinor A. A new framework for predicting and understanding flowering time for crop breeding. Plants People Planet. 2023;6(1):197–209. doi: 10.1002/ppp3.10427

[11] Muniswamaiah M, Agerwala T, Tappert CC. Big Data and Data Visualization Challenges. In: 2023 IEEE International Conference on Big Data (BigData). IEEE; 2023. pp. 6227–9.

[12] Song H, Szafir DA. Where’s my data? Evaluating visualizations with missing data. IEEE Trans Vis Comput Graph. 2018. doi: 10.1109/TVCG.2018.2864914 30136964

[13] Kowarik A, Templ M. Imputation with the R Package VIM. J Stat Soft. 2016;74(7). doi: 10.18637/jss.v074.i07

[14] Austin PC, White IR, Lee DS, van Buuren S. Missing data in clinical research: a tutorial on multiple imputation. Can J Cardiol. 2021;37(9):1322–31. doi: 10.1016/j.cjca.2020.11.010 33276049 PMC8499698

[15] Sterne JAC, White IR, Carlin JB, Spratt M, Royston P, Kenward MG, et al. Multiple imputation for missing data in epidemiological and clinical research: potential and pitfalls. BMJ. 2009;338:b2393. doi: 10.1136/bmj.b2393 19564179 PMC2714692

[16] Ke G, Meng Q, Finley T, Wang T, Chen W, Ma W, et al. LightGBM: A highly efficient gradient boosting decision tree. Advances in Neural Information Processing Systems. 2017;30.

[17] Enders CK. Applied missing data analysis. Guilford Publications; 2022.

[18] Fernstad SJ. To identify what is not there: A definition of missingness patterns and evaluation of missing value visualization. Inform Visualiz. 2018;18(2):230–50. doi: 10.1177/1473871618785387

[19] Weberpals J, Raman SR, Shaw PA, Lee H, Hammill BG, Toh S, et al. smdi: an R package to perform structural missing data investigations on partially observed confounders in real-world evidence studies. JAMIA Open. 2024;7(1):ooae008. doi: 10.1093/jamiaopen/ooae008 38304248 PMC10833461

[20] Ruddle RA. Using Well-Known Techniques to Visualize Characteristics of Data Quality. In: VISIGRAPP (3: IVAPP). 2023. pp. 89–100.

[21] Ruddle RA, Adnan M, Hall M. Using set visualisation to find and explain patterns of missing values: a case study with NHS hospital episode statistics data. BMJ Open. 2022;12(11):e064887. doi: 10.1136/bmjopen-2022-064887 36410820 PMC9680176

[22] Elobeid MA, Padilla MA, McVie T, Thomas O, Brock DW, Musser B, et al. Missing data in randomized clinical trials for weight loss: scope of the problem, state of the field, and performance of statistical methods. PLoS One. 2009;4(8):e6624. doi: 10.1371/journal.pone.0006624 19675667 PMC2720539

[23] Baena-Miret S, Reverter F, Vegas E. A framework for block-wise missing data in multi-omics. PLoS One. 2024;19(7):e0307482. doi: 10.1371/journal.pone.0307482 39042603 PMC11265675

[24] Rippe RCA, Merkelbach I. Planned missing data in early literacy interventions: a replication study with an additional gold standard. PLoS One. 2021;16(3):e0249175. doi: 10.1371/journal.pone.0249175 33780486 PMC8007029

[25] Zhang Z. Missing data exploration: highlighting graphical presentation of missing pattern. Ann Transl Med. 2015;3(22):356. doi: 10.3978/j.issn.2305-5839.2015.12.28 26807411 PMC4701517

[26] Adnan M, Nguyen PH, Ruddle RA, Turkay C. Visual analytics of event data using multiple mining methods. In: EuroVis Workshop on Visual Analytics (EuroVA) 2019. The Eurographics Association; 2019. pp. 61–5.

[27] Oberman H. ggmice: Visualizations for ’mice’ with ’ggplot2’. 2023. Available from: https://cran.r-project.org/web/packages/ggmice/

[28] Ward M, Xie Z, Yang D, Rundensteiner E. Quality-aware visual data analysis. Comput Stat. 2011;26(4):567–84. doi: 10.1007/s00180-010-0226-0

[29] Kandel S, Parikh R, Paepcke A, Hellerstein JM, Heer J. Profiler: Integrated statistical analysis and visualization for data quality assessment. In: Proceedings of the International Working Conference on Advanced Visual Interfaces. 2012. pp. 547–54.

[30] Trifacta Software. Trifacta Wrangler. 2018. Available from: https://www.trifacta.com/

[31] Ruddle R, Hall M. Using Miniature Visualizations of Descriptive Statistics to Investigate the Quality of Electronic Health Records. In: Proceedings of the 12th International Joint Conference on Biomedical Engineering Systems and Technologies-Volume 5: HEALTHINF. SciTePress; 2019. pp. 230–8.

[32] Alsufyani S, Forshaw M, Del Din S, Yarnall A, Rochester L, Fernstad SJ. Multi-level visualization for exploration of structures in missing data. Proceedings of Computer Graphics and Visual Computing (CGVC). 2024.

[33] Jiménez E, Macías R. Graphical Tools for Visualization of Missing Data in Large Longitudinal Phenomena. In: Computer Graphics Forum. vol. 41. Wiley Online Library; 2022. pp. 438–52.

[34] Conway JR, Lex A, Gehlenborg N. UpSetR: an R package for the visualization of intersecting sets and their properties. Bioinformatics. 2017;33(18):2938–40. doi: 10.1093/bioinformatics/btx364 28645171 PMC5870712

[35] Lex A, Gehlenborg N, Strobelt H, Vuillemot R, Pfister H. UpSet: visualization of intersecting sets. IEEE Trans Vis Comput Graph. 2014;20(12):1983–92. doi: 10.1109/TVCG.2014.2346248 26356912 PMC4720993

[36] Nothman J. UpSetPlot. 2022. Available from: https://pypi.org/project/UpSetPlot/

[37] Tierney N, Cook D, McBain M, Fay C. naniar: Data Structures, Summaries, and Visualisations for Missing Data. 2018. Available from: https://cloud.r-project.org/web/packages/naniar/index.html

[38] Wang G, Ma M, Jiang L, Chen F, Xu L. Multiple imputation of maritime search and rescue data at multiple missing patterns. PLoS One. 2021;16(6):e0252129. doi: 10.1371/journal.pone.0252129 34143787 PMC8213137

[39] Alsallakh B, Micallef L, Aigner W, Hauser H, Miksch S, Rodgers P. The state‐of‐the‐art of set visualization. Comput Graph Forum. 2015;35(1):234–60. doi: 10.1111/cgf.12722

[40] Ruddle RA, Hama L, Wochner P, Strickson OT. SetVis: visualizing large numbers of sets and intersections. J Open Source Softw. 2024;9(103):6925. doi: 10.21105/joss.06925

[41] Ragan ED, Endert A, Sanyal J, Chen J. Characterizing provenance in visualization and data analysis: an organizational framework of provenance types and purposes. IEEE Trans Vis Comput Graph. 2016;22(1):31–40. doi: 10.1109/TVCG.2015.2467551 26340779

[42] Simmhan YL, Plale B, Gannon D, et al. A survey of data provenance techniques. Bloomington IN: Computer Science Department, Indiana University; 2005;47405:69.

[43] Bors C, Gschwandtner T, Miksch S. Capturing and visualizing provenance from data wrangling. IEEE Comput Graph Appl. 2019;39(6):61–75. doi: 10.1109/MCG.2019.2941856 31581076

[44] Callahan SP, Freire J, Santos E, Scheidegger CE, Silva CT, Vo HT. VisTrails: visualization meets data management. In: Proceedings of the 2006 ACM SIGMOD International Conference on Management of Data; 2006. pp. 745–7.

[45] Nguyen PH, Xu K, Bardill A, Salman B, Herd K, Wong BW. SenseMap: Supporting browser-based online sensemaking through analytic provenance. In: 2016 IEEE Conference on Visual Analytics Science and Technology (VAST). IEEE; 2016. pp. 91–100.

[46] Strack B, DeShazo JP, Gennings C, Olmo JL, Ventura S, Cios KJ, et al. Impact of HbA1c measurement on hospital readmission rates: analysis of 70,000 clinical database patient records. BioMed Res Int. 2014;2014. 10.1155/2014/781670 PMC3996476 24804245

[47] Herbert A, Wijlaars L, Zylbersztejn A, Cromwell D, Hardelid P. Data resource profile: hospital episode statistics admitted patient care (HES APC). Int J Epidemiol. 2017;46(4):1093–1093i. doi: 10.1093/ije/dyx015 28338941 PMC5837677

[48] Wang Q, Laramee RS. EHR STAR: The state-of-the-art in interactive EHR visualization. In: Computer Graphics Forum. vol. 41. Wiley Online Library; 2022. pp. 69–105.

[49] NHS-Digital. HES Data Dictionary - Admitted Patient Care. 2018.

[50] Holten D. Hierarchical edge bundles: visualization of adjacency relations in hierarchical data. IEEE Trans Vis Comput Graph. 2006;12(5):741–8. doi: 10.1109/TVCG.2006.147 17080795

